# A gastruloid model of the interaction between embryonic and extra-embryonic cell types

**DOI:** 10.1177/20417314221103042

**Published:** 2022-06-11

**Authors:** Noémie MLP Bérenger-Currias, Maria Mircea, Esmée Adegeest, Patrick R van den Berg, Marleen Feliksik, Mazène Hochane, Timon Idema, Sander J Tans, Stefan Semrau

**Affiliations:** 1 Department of Physics, Leiden University, Leiden, The Netherlands; 2 Delft University of Technology, Department of Bionanoscience, Kavli Institute of Nanoscience, Delft, The Netherlands; 3 AMOLF, Amsterdam, The Netherlands

**Keywords:** Gastruloids, neuroepithelium, single-cell transcriptomics, stem cell engineering

## Abstract

Stem-cell derived in vitro systems, such as organoids or embryoids, hold great potential for modeling in vivo development. Full control over their initial composition, scalability, and easily measurable dynamics make those systems useful for studying specific developmental processes in isolation. Here we report the formation of gastruloids consisting of mouse embryonic stem cells (mESCs) and extraembryonic endoderm (XEN) cells. These XEN-enhanced gastruloids (XEGs) exhibit the formation of neural epithelia, which are absent in gastruloids derived from mESCs only. By single-cell RNA-seq, imaging, and differentiation experiments, we demonstrate the neural characteristics of the epithelial tissue. We further show that the mESCs induce the differentiation of the XEN cells to a visceral endoderm-like state. Finally, we demonstrate that local inhibition of WNT signaling and production of a basement membrane by the XEN cells underlie the formation of the neuroepithelial tissue. In summary, we establish XEGs to explore heterotypic cellular interactions and their developmental consequences in vitro.

## Introduction

**Figure 1.**
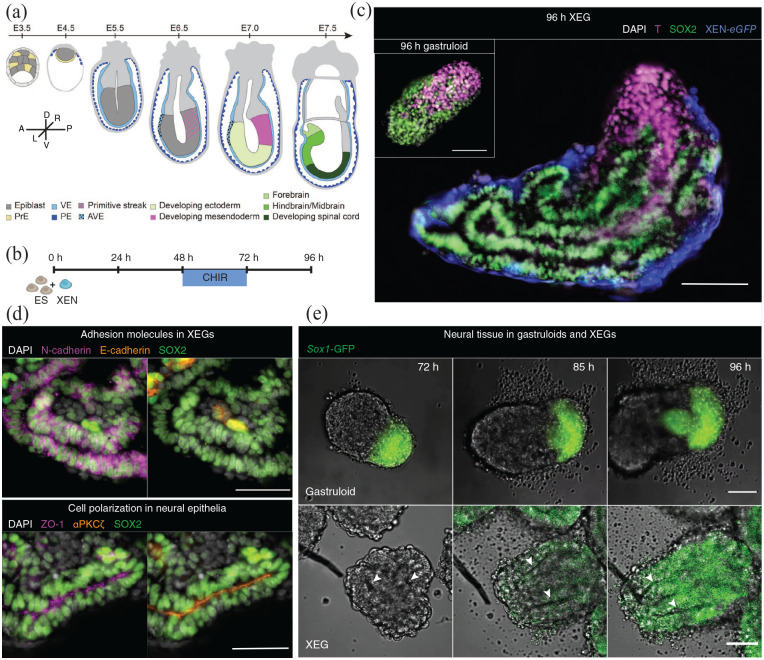
XEN cells induce neuroepithelial structures in XEN enhanced gastruloids. (a) Schematic of early mouse embryonic development. Tissues discussed in this manuscript are indicated with color. A: anterior; D: dorsal; L: left; P: posterior; R: right; V: ventral. (b) Schematic of the culture protocol: at 0 h, 200 cells (150 ESCs and 50 XEN cells) were aggregated; CHIR99021 was added between 48 and 72 h after cell seeding to activate the WNT pathway; cell aggregates were cultured until 96 h. (c) T and SOX2 expression at 96 h in XEGs. Inset: Aggregate resulting from the standard gastruloid protocol (without XEN cells) at 96 h. Z-projections of wholemount immunostaining. Scale bars: 100 µm. (d) Expression of SOX2, E-cadherin, N-cadherin, ZO-1 and αPKCζ in XEGs at 96 h (immunostaining of cryosections). Scale bar: 50 µm. (e) Live-cell imaging of SOX1 expression in a gastruloid (top panels, scale bar: 20 µm) and a XEG (bottom panels, scale bar: 50 µm), grown with *Sox1-*GFP mESCs (see Supplemental Videos 3–8). In all images, a single z-plane is shown. The arrows indicate epithelial structures. (c and d) Cell nuclei were stained with DAPI.

Multicellular in vitro systems have become a major focus of biology and bioengineering over the last few years. Stem cell-derived systems, such as embryoids and organoids show complex organization and have the potential to serve as models for in vivo development.^1–3^ Among the most prominent examples of such model systems are gastruloids. These aggregates of mouse or human embryonic stem cells (ESCs) recapitulate elements of embryonic development, such as body axis formation and extension.^4–9^ Notably, gastruloids do not contain extraembryonic cells, which provide numerous signaling inputs during gastrulation in vivo.^10^ The remarkable self-organizing capabilities of ESCs thus raise questions about the precise role of extraembryonic tissues in gastrulation. Here, we will focus on the extraembryonic endoderm, which derives from the primitive endoderm (PrE) in vivo. At the blastocyst stage, prior to implantation of the embryo in the uterine wall, the PrE overlays the developing epiblast, which gives rises to all embryonic tissues (see Figure 1(a) for a schematic of early mouse development). Subsequently, the PrE differentiates into the Parietal Endoderm (PE), which covers the inside of the blastocoel cavity^11^ and the Visceral Endoderm (VE), which surrounds the embryo until the formation of the visceral yolk sac and integration of some VE cells in the embryonic gut.^12,13^ Another subpopulation of the VE, the Anterior Visceral Endoderm (AVE) is involved in the establishment of the embryo’s body axes.^14,15^ In this study, we set out to develop an in vitro model system for the interaction between the extraembryonic endoderm and the gastrulating embryo. As a proxy for the extraembryonic endoderm in vivo, we used XEN cells, which can be derived from the PrE cells in blastocysts. XEN cells have been previously incorporated in embryoid systems^16–20^ that model the earliest stages of development. Here, we wanted to explore, whether the role of the extraembryonic endoderm in gastrulation can be modeled by adding XEN cells to the gastruloid model system. Below, we report that aggregates of mESCs and XEN cells can produce columnar neural epithelia. Using multiple markers, perturbation of the signaling pathways that play a role in neural development in vivo, and further differentiation to neural organoids, we confirmed that the epithelial structures indeed have neural characteristics. By single-cell RNA-seq, we identified differences in composition and molecular profiles between our new model system and regular gastruloids. We then established that a majority of XEN-derived cells become visceral endoderm-like due to co-differentiation with the mESCs. Finally, we showed that XEN cells promote epithelia formation by local, DKK1 mediated, WNT inhibition, as well as through production of a basement membrane. Our study thus highlights the complex interplay between embryonic and extraembryonic cells and explores possible mechanisms underlying their interaction.

## Results

### XEN cells induce neuroepithelial structures in XEN enhanced gastruloids

We first implemented the original mouse gastruloid protocol,^4^ in which mESCs are aggregated in N2B27 media and exposed to a 24 h pulse of CHIR99021 (CHIR), which activates the WNT pathway. After 96 h, this protocol results in elongated gastruloids. As reported before,^4–6^ 96 h gastruloids contained localized compartments, marked by either Brachyury (T) or SOX2, (Figure 1(c), inset). These compartments are believed to resemble early in vivo mesendodermal (T) or neural progenitor (SOX2) cell types. Starting from the gastruloid protocol, we developed a new system by aggregating mESCs and XEN cells, keeping all other experimental conditions the same (Figure 1(b)). We call our mixed aggregates “XEN Enhanced Gastruloids” (XEGs). Like gastruloids, 96 h XEGs showed an elongated morphology and localized T-positive and SOX2-positive compartments. However, unlike in gastruloids, SOX2-positive cells in XEGs were organized in columnar epithelia surrounding one or several lumina (Figure 1(c)).

Expression of the broadly expressed neural marker SOX2 and the striking morphology suggested that the observed structures resemble neural epithelia. The lack of pluripotency marker expression (Supplemental Figure 1(a)) excluded that the structures were formed by remaining undifferentiated cells. The presence of N-cadherin and absence of E-cadherin in the epithelia (Figure 1(d), top) is consistent with the known switch from E- to N-cadherin during neural differentiation in vivo^21^ and in vitro.^22^ We could also observe that the epithelial cells were polarized and expressed apical markers ZO-1 and aPKC (Figure 1(d), bottom), consistent with neural epithelia in vivo.^23^ Finally, we detected the neural progenitor markers PAX6 and NKX6.1^24^ in a subpopulation of epithelial cells (Supplemental Figure 1(b)). Combined, these results suggest that the observed structures in XEGs have the characteristics of neural epithelia.

To understand how these structures formed, we used time-lapse microscopy of developing XEGs. Around 48 h after seeding, cells formed rosette-like shapes (Supplemental Figure 1(c), Supplemental Video 1), which resembled structures found in Matrigel-embedded mESCs^25,26^ and indicated a mesenchymal-epithelial transition. Subsequently, a columnar epithelium was formed. Then, lumina opened at different places and merged between 48 and 72 h (Supplemental Figure 1(d), Supplemental Video 2). During the final 24 h, the epithelium kept extending and differentiated further, as revealed, by the expression of the neural progenitor marker SOX1^27^ (Figure 1(e), bottom; Supplemental Videos 3–5). A SOX1 positive cell population also appeared in gastruloids within the same time frame, but, importantly, remained unorganized (Figure 1(e), top; Supplemental Videos 6–8).

To explore the robustness of the protocol and identify optimal conditions for the formation of epithelial structures, we tested different ratios of mESCs and XEN cells (Supplemental Figure 1(e) and (f)). Interestingly, even the smallest proportion of XEN cells tested (1:5), was able to induce some epithelia formation. On the other hand, elongation and symmetry breaking were inhibited when the proportion of XEN cells exceeded 1:2. A ratio of 1:3 gave optimal results, with the concurrence of SOX2-positive epithelia and T-positive cells in nearly all aggregates.

### Neuroepithelial cells in XEGs are heterogeneous and show further specification

**Figure 2.**
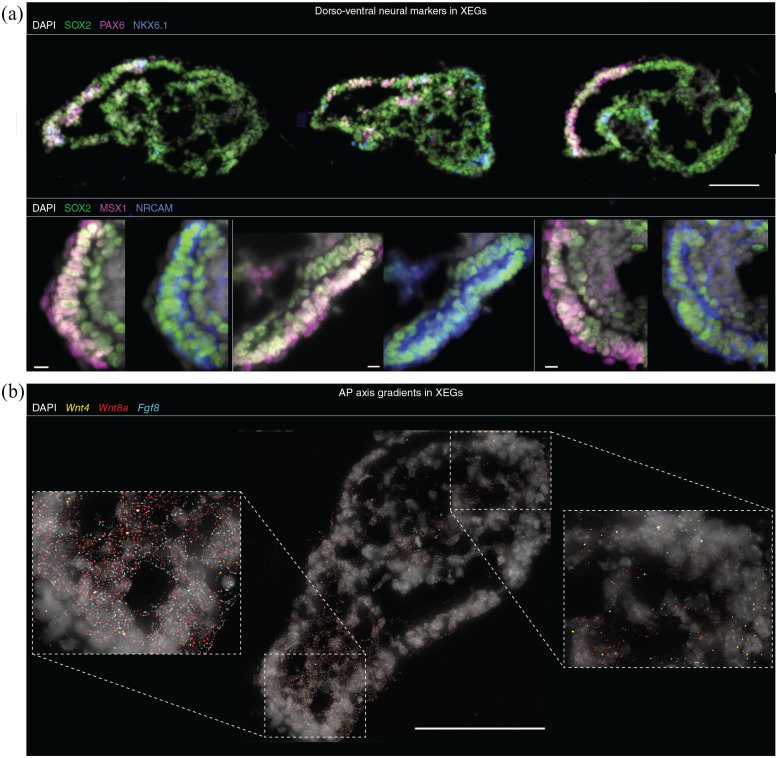
Neural epithelia in XEGs are heterogeneous and contain subpopulations with dorsal or ventral characteristics. (a) Expression of dorsal (PAX6, MSX1) and ventral (NKX6.1) neural tube markers and a neural cell adhesion molecule (NRCAM) in 96 h XEGs (immunostaining of sections). Top, expression of PAX6 and NKX6.1. Scale bar: 100 µm. Bottom, zoomed pictures of neural epithelia showing the expression of MSX1 and NRCAM. Scale bars: 20 µm. (b) *Wnt4, Wnt8a*, and *Fgf8* expression in XEGs at 96 h, visualized by smFISH on sections. Each diffraction limited dot is a single mRNA molecule. Scale bar: 100 µm. (a and b) Cell nuclei were stained with DAPI.

To establish whether the neuroepithelial cells are a homogeneous population of progenitors or have undergone further specification, we carried out additional immunostaining. In subpopulations of cells, we observed the expression of PAX6, MSX1, and ASCL1 (Figure 2(a), Supplemental Figure 1(b)), which can be found in dorsal progenitors in the developing neural tube.^28^ Notably, these markers were localized close to the XEN-derived cells at the outside of the XEGs. By contrast, the ventral marker NKX6.1 was found only sporadically and did not show any preferential spatial localization (Figure 2(a)). Finally, the neural adhesion molecule NRCAM was ubiquitously expressed in epithelial cells, further supporting their neural character (Figure 2(a)).

We also attempted to establish a possible specification related to the anteroposterior axis in vivo. Using single-molecule FISH, we observed the expression of *Wnt4, Wnt8a*, and *Fgf8* as gradients along the long axis of XEGs (Figure 2(b)), which resembled similar anteroposterior gradients found in vivo.^29^ However, important canonical markers of the most anterior part of the embryo (OTX2, LEFTY1, EN1, ZIC1) could not be detected in XEGs (data not shown). Taken together, our measurements indicated that neuroepithelial cells in XEGs are heterogeneous and contain subpopulations that might correspond to either dorsal or ventral neural progenitors.

### Signaling perturbation experiments and further differentiation support the neuroepithelial character

**Figure 3.**
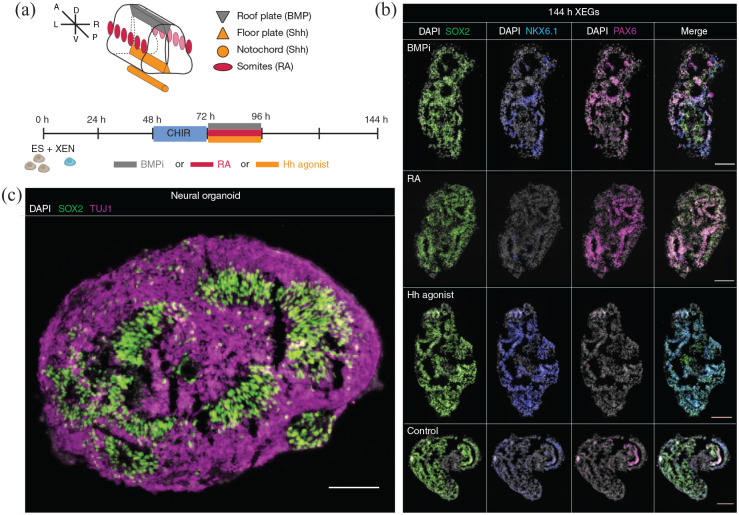
Signaling perturbation experiments and continued differentiation confirm neural character. (a) Top: schematic of signaling sources patterning the developing neural tube in vivo. A: anterior; D: dorsal; L: left; P: posterior; R: right; V: ventral. Bottom: time line of the signaling experiments. XEGs were treated from 72 to 96 h, with either BMP pathway inhibitor (BMPi), retinoic acid (RA), or hedgehog pathway agonist (Hh agonist). The XEGs were then allowed to grow for an additional 48 h before staining. (b) Expression of SOX2, NKX6.1, and PAX6 in XEGs at 144 h, treated with the indicated factors (immunostaining of sections). *n* = 3 experiments. Scale bars: 100 µm. (c) Expression of SOX2 and TUJ1 in XEGs, 8 days after cell seeding, differentiated according to a cerebral organoid protocol for 4 days (immunostaining of sections). Scale bar: 100 µm. (b and c) Cell nuclei are stained with DAPI.

To further characterize the neuroepithelial structures, we tested how they respond to signaling inputs found in vivo. Specifically, we explored the response to BMP pathway inhibition, as well as Sonic Hedgehog (Shh) and retinoic acid (RA) pathway activation (Figure 3(a) and (b)). BMP signaling is known to prevent premature neural specification^30^ and to be involved in dorsal patterning of the neural tube.^31^ In XEGs, BMP inhibition resulted in an increased number of cells expressing the neural progenitor markers SOX2 and PAX6, as well as NKX6.1, which is expressed in ventral progenitors in the developing neural tube. Sonic hedgehog, produced in vivo by the notochord and the floor plate (see schematic in Figure 3(a)), is known to be necessary for the patterning of the ventral part of the neural tube.^32^ The activation of the Hedgehog signaling pathway led to a higher frequency of cells expressing a ventral marker (NKX6.1) in XEGs. RA, involved in anteroposterior and dorsoventral patterning,^33^ strongly increased the number of cells expressing PAX6, which is expressed in dorsal progenitors in the neural tube in vivo.^34^ The neuroepithelial structures in XEGs thus responded to signaling inputs as expected from in vivo development.

To test the developmental potential of the neural progenitors further, we sought to differentiate them to more advanced states. Within 4 days of additional culture in cerebral organoid differentiation media,^35^ XEGs developed a layered organization of neural progenitors (SOX2+/PAX6+) and neurons (TUJ1+/CTIP2+/PAX2+), surrounding cavities, reminiscent of the organization of the developing dorsal spinal cord^28,36^ (Figure 3(c), Supplemental Figure 2(a)–(c)). Interestingly, we also observed a population of cells expressing the endothelial marker CD31 (Supplemental Figure 2(d)). This might indicate that non-neural cells remained and might have differentiated further. Those CD31+ cells could specifically represent an early stage of vasculature. Taken together, the signaling perturbation and differentiation experiments confirmed the neural potential of the epithelia.

### Single-cell RNA-seq reveals the transcriptional profiles of XEG cells

**Figure 4.**
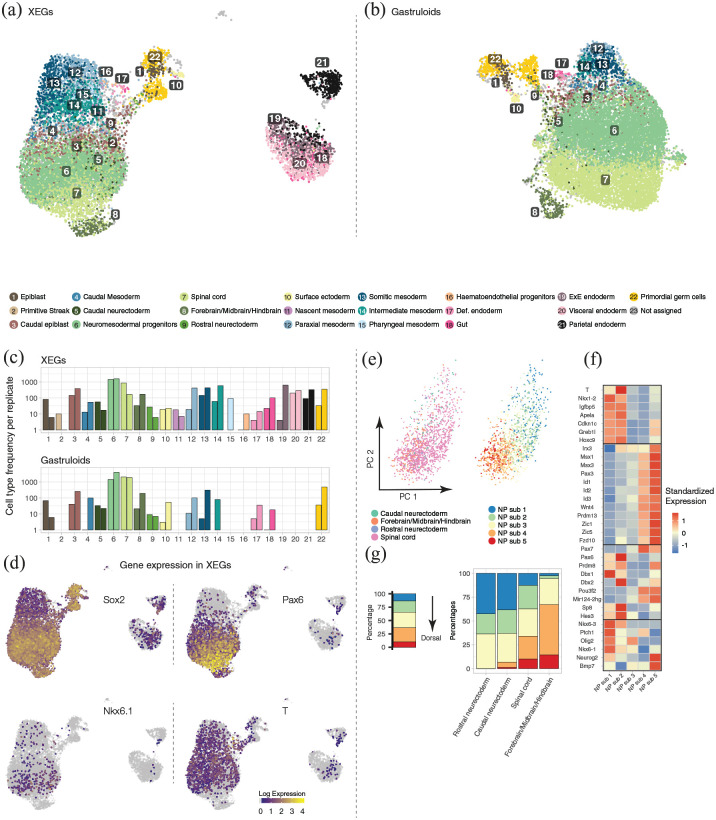
Single-cell RNA-seq reveals the transcriptional profiles of XEG cells. (a and b) UMAP of cells in XEGs and gastruloids colored by cell type based on the mapping to in vivo data shown in Supplemental Figure 4(a) and (b). (c) Cell type frequencies for both replicates in XEGs and gastruloids. (d) *Sox2, Pax6, Nkx6.1*, and *T* log-expression levels indicated by color in UMAPs of XEGs. (e) Principal Component Analysis of neural ectoderm-like cells in XEGs. Left: Colored by cell type based on the mapping to in vivo data. Right: Colored by neural progenitor subtypes (NP sub) found by sub-clustering. (f) Heatmap of standardized expression of dorsoventral markers in the sub-clusters of neural ectoderm-like cells shown in (e). (g) Relative frequency (percentage) of sub-clusters. Left: For all neural ectoderm-like cells. Right: Per cell type (based on mapping to in vivo data).

Having focused on the most striking, morphological difference between gastruloids and XEGs, we wanted to take a more comprehensive approach to reveal additional differences between the two model systems. To that end, we used single-cell RNA-sequencing (scRNA-seq) (Supplemental Figure 3(a)–(e)). By mapping the data to single-cell transcriptomes of mouse embryos from E6.5 to E8.5^37^ (Supplemental Figure 4(a) and (b)) we classified the transcriptional identity of the cells (Figure 4(a) and (b)). Except for the least abundant cell types, the distribution of cell types was consistent across two biological replicates (Figure 4(c)). Expression of known markers confirmed the classification by mapping to in vivo data (Supplemental Figure 4(c), Supplemental Table 1). Most cell types belonged to the E8.0 or E8.5 embryo (Supplemental Figure 4(d)), which might indicate that in vitro differentiation proceeded roughly with the same speed as in vivo development.

Neuromesodermal progenitors (NMPs) and spinal cord-like cells were the most abundant in both model systems (Figure 4(c)). Gastruloids thus already contain cells of the neural lineage, which, however, seem to lack organization (Figure 1(c), inset). To identify the cells forming epithelial structures in XEGs, we used the neural markers *Sox2, Pax6*, and *Nkx6.1*,^24^ which we had detected by immunostaining (Supplemental Figure 1(b)). We found these markers to be co-expressed in cells classified as “spinal cord” and “brain” in the scRNA-seq data (Figure 4(d), Supplemental Figure 4(e)), confirming their neural ectoderm identity. While NMPs also expressed *Sox2* and *Nkx6.1*, neuroepithelial structures were clearly distinguishable by the presence of *Pax6* and the absence of *T*.

We next asked, whether there are any subpopulations in the neural ectoderm-like cells, as hinted at by our immunostaining results (Figure 2). Differential gene expression analysis between spinal cord-like cells and other cells in XEGs identified markers of both the dorsal and ventral neural tube (Supplemental Figure 5(a), Supplemental Table 2). A comparison between XEGs and gastruloids revealed that neural ectoderm-like cells (including spinal cord and clusters identified as neuroectoderm or brain) expressed more dorsal markers in XEGs (Supplemental Figure 5(b), Supplemental Table 3). This dorsal identity was confirmed by mapping the neural ectoderm-like cells to single-cell expression profiles of in vivo neural tube^36^ (Supplemental Figure 5(c)). The majority of neural ectoderm-like cells from XEGs turned out to be more similar to dorsal progenitors in vivo. To reveal subpopulations, we clustered the neural ectoderm-like cell using a curated list of genes that are dorsoventral axis markers in the developing neural tube^28,36,38^ (Figure 4(e)). This analysis resulted in five clusters with distinct dorsoventral characteristics (Figure 4(f)). Two clusters (1 and 2) had a more ventral identity, two clusters (4 and 5) had dorsal transcriptional characteristics and cluster 3 expressed both dorsal and ventral markers at a low level. Roughly one third of the cells had a dorsal identity (Figure 4(g)), which is qualitatively consistent with our immunostaining results (Figure 2). We repeated this analysis on integrated neural ectoderm-like cells from gastruloids and XEGs. Strikingly, the clusters with clear dorsal characteristics were almost exclusively comprised of XEG cells (Supplemental Figure 5(d)). XEN-derived cells thus seem to promote dorsal specification in a subpopulation of neural ectoderm-like cells. Judged by the expression of *Hox* genes (Supplemental Figure 5(e)), there was no overt difference in anterior-posterior characteristics between the neural ectoderm-like clusters in XEGs. However, scRNA-seq did confirm the heterogeneous expression of *Fgf8, Wnt4*, and *Wnt8a* (Supplemental Figure 5(f)) we had observed by smFISH (Figure 2(b)), hinting at additional subpopulations related to the anteroposterior axis in vivo.

Overall, both model systems showed a diverse cell type distribution, also comprising a variety of mesodermal cell types. Thus, XEG cells are not globally biased toward the neural fate, as occurring in other protocols for induction of neural epithelia.^26,39,40^ On the contrary, XEGs even contained a bigger proportion of mesoderm-like cells, compared to gastruloids (Figure 4(c), Supplemental Figure 6(a)). While paraxial, intermediate and somitic mesoderm-like clusters were present in both model systems, only XEGs contained cells transcriptionally resembling primitive streak, nascent mesoderm, pharyngeal mesoderm, and hematoendothelial progenitors. To confirm the presence of mesodermal cell types in XEGs, we focused on two genes, *Tbx6* and *Pax2*, which are markers of nascent and intermediate mesoderm, respectively. Our single-cell RNA-seq data showed expression of both genes in subpopulations of XEG cells (Supplemental Figure 6(b)) and immunostaining confirmed their presence (Supplemental Figure 6(c)). However, we did not observe any tissue-level organization of those cells in XEGs. Taken together, these results suggest that the XEN-derived cells in XEGs also have an effect on the mesodermal cell population.

### Most XEN cells become VE-like in XEGs

**Figure 5.**
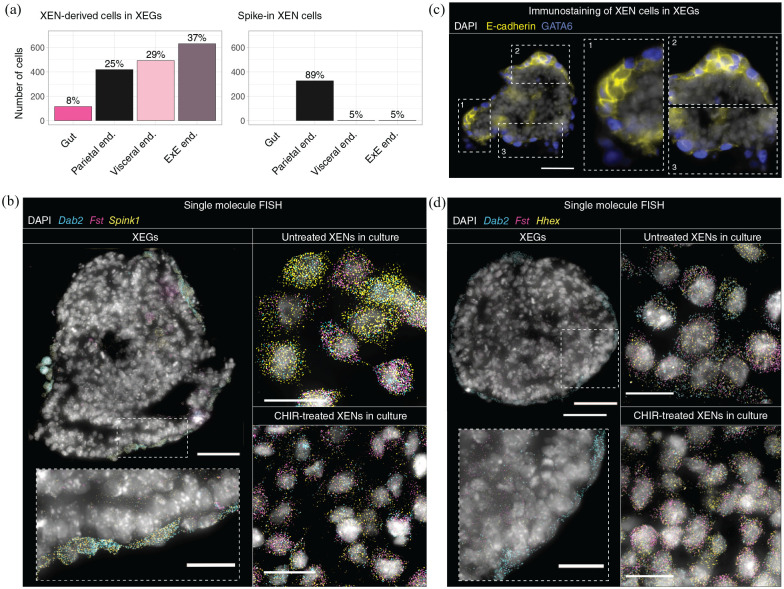
Most XEN cells become VE-like in XEGs. (a) Left, cell types of XEN-derived cells in XEGs. Cells were classified as gut, PE (“parietal end.”), embryonic VE (“visceral end.”) or extraembryonic VE (“ExE end.”) by mapping to the data set from Pijuan-Sala et al.^37^ Right, cell types of spiked-in XEN cells. (b) Dab2, *Spink1*, and *Fst* expression in a section of an XEG at 96 h (left, scale bar: 50 µm), in XEN cells cultured under standard maintenance conditions (top right, scale bar: 20 µm) and in XEN cells treated with CHIR according to the XEG protocol (bottom right, scale bar: 20 µm). Expression was visualized by smFISH. Each diffraction limited dot is a single mRNA molecule. (c) Expression of E-cadherin in XEGs at 96 h (immunostaining of sections). XEN cells were localized by expression of GATA6. Scale bars: 50 µm. (d) Dab2, *Fst*, and *Hhex* expression in a section of an XEG at 96 h (left, scale bar: 50 µm), in XEN cells cultured under standard maintenance conditions (top right, scale bar: 20 µm) and in XEN cells treated with CHIR according to the XEG protocol (bottom right, scale bar: 20 µm). Expression was visualized by smFISH. Each diffraction limited dot is a single mRNA molecule. (b–d) Cell nuclei were stained with DAPI. The dashed boxes are shown at a higher magnification in the insets.

Compared to gastruloids, XEGs additionally contained extraembryonic endoderm cell types (Figure 4(c)). By using GFP-expressing XEN cells in XEGs (Supplemental Figure 7(a)), we established that those cell types were exclusively differentiated from XEN cells. By comparison to undifferentiated XEN cells, which were spiked into the scRNA-seq samples, we studied the transcriptional changes in XEN-derived cells. Undifferentiated XEN cells mostly mapped to PE^37^ (Figure 5(a), Supplemental Figure 7(b)), consistent with a previous study.^41,42^ Their derivatives in XEGs mapped to multiple kinds of extraembryonic endoderm: PE, embryonic VE and extraembryonic VE. Interestingly, some also mapped to gut, reminiscent of the contribution of VE to the gut in vivo.^12,43^ The identification of those cell types was confirmed by mapping our scRNA-seq data to an endoderm-focused scRNA-seq dataset^43^ (Supplemental Figure 7(c) and (d)). Quantification revealed that, on average, 8% of the initially PE-like XEN cells acquired a gut-like and 66% a VE-like transcriptomic profile (29% embryonic VE, 37% extraembryonic VE) when co-cultured in XEGs. However, 25% retained a PE-like transcriptome (Figure 5(a)). Differential gene expression analysis between undifferentiated XEN cells and XEN-derivatives revealed several differences (Supplemental Figure 7(e), Supplemental Table 4). PE markers were less expressed in XEN-derivatives, while VE markers were highly expressed, suggesting that most XEN cells differentiate from a PE to a VE-like state in XEGs. To validate this finding experimentally, we performed single-molecule FISH of *Dab2, Fst*, and *Spink1* (Figure 5(b)). *Dab2* is a pan-extraembryonic endoderm marker,^44^ which is exclusively expressed in undifferentiated XEN cells and XEN-derived cell types in our scRNA-seq data set (Supplemental Figure 4(c)). Within the extraembryonic endoderm, *Fst* is expressed in the PE,^45^ whereas *Spink1* is found in the VE.^46^ The smFISH measurement showed that XEN-derived cells in XEGs only expressed *Dab2* and *Spink1*, while undifferentiated XEN cells broadly co-expressed all markers. Some XEN cells in XEGs were also highly expressing E-cadherin, known to be expressed in VE^47^ (Figure 5(c)). However, the more anterior VE marker *Hhex*^48^ was not detected by single-molecule FISH (Figure 5(d)). Exposing undifferentiated XEN cells to CHIR in the same way as XEGs did not cause differentiation (Figure 5(d)), which suggests that the interaction with mESCs plays a role. Cell-cell communication analysis of our scRNA-seq data with CellPhoneDB^49^ suggested that mESCs and some mesodermal cell types in XEGs signal to the XEN-derived cells via the BMP4 pathway (Supplemental Figure 7(f)). This result is consistent with a previous study showing the differentiation of XEN cells in monolayer culture to a VE-like state with BMP.^50^

Taken together, these results suggest that the mESCs or their derivatives induce the differentiation of the XEN-derived cells in XEGs. While undifferentiated XEN cells have both PE and VE characteristics, the majority (66%) of these cells becomes more VE-like. This effect is possibly mediated by BMP signaling originating in the mESCs.

### XEN cells guide symmetry breaking by local inhibition of WNT signaling

**Figure 6.**
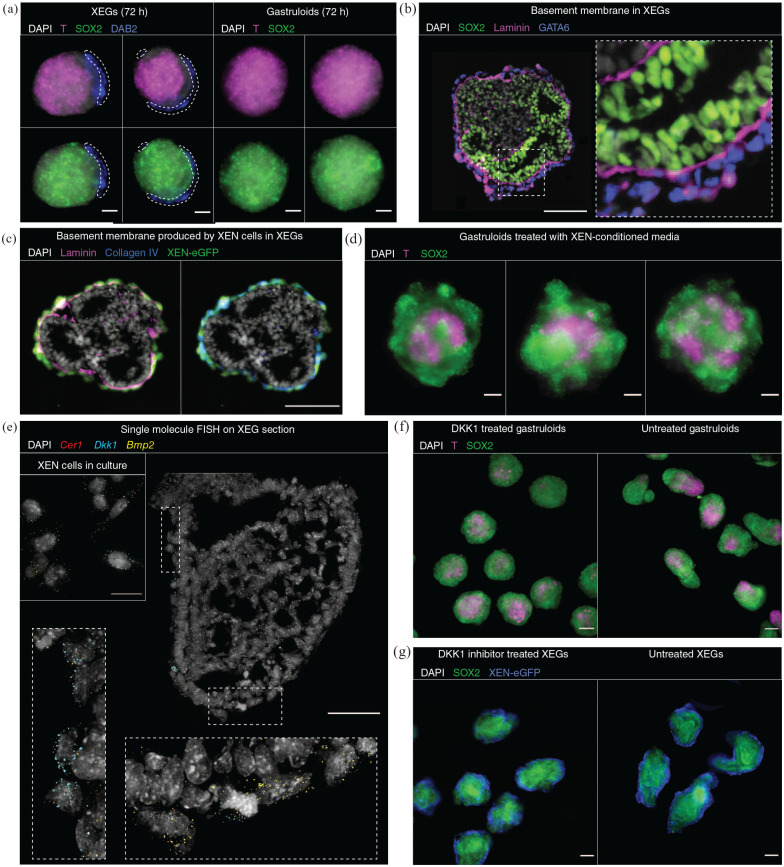
XEN cells guide symmetry breaking by local WNT inhibition and contribution to a basement membrane. (a) T and SOX2 expression in XEGs (left) and gastruloids (right) at 72 h (z-projection of whole mount immunostaining). XEN cells were localized by expression of DAB2 and are indicated by a dashed outline. (b) Expression of SOX2 and laminin in XEGs at 96 h (immunostaining of sections). XEN cells were localized by expression of GATA6. The dashed box is shown at a higher magnification in the inset. Scale bar: 50 µm. (c) Expression of collagen IV and laminin in XEGs at 96 h (immunostaining of sections). XEN cells were localized by expression of H2B-GFP. Scale bar: 100 µm. (d) T and SOX2 expression in gastruloids grown in XEN-conditioned media at 96 h (z-projection of whole mount immunostaining). (e) Cer1, Dkk1, and *Bmp2* expression in a section of a XEG at 96 h (scale bar: 50 µm) or XEN cells cultured under standard maintenance conditions (inset, scale bar: 20 µm). Expression was visualized by smFISH. Each diffraction limited dot is a single mRNA molecule. The dashed boxes are shown at a higher magnification in the insets. (f) T and SOX2 expression in 96 h gastruloids treated with 200 ng/mL DKK1 and untreated (z-projection of whole mount immunostaining). Scale bars: 100 µm. (g) T and SOX2 expression in 96 h XEGs treated with 0.25 µM DKK1 inhibitor WAY-262611 and untreated (z-projection of whole mount immunostaining). Scale bars: 100 µm. (a–g) Cell nuclei were stained with DAPI.

Although XEN-derived cells in XEGs did not express canonical AVE markers (Supplemental Figure 4(c)), we were wondering if they might effectively carry out an AVE-like function. XEN cells always formed the outermost layer (Figure 1(c), Supplemental Figure 1(f)), resembling in vivo organization. Focusing on XEGs partially covered with XEN cells, we observed that epithelial structures were always adjacent to the XEN cells, while the T-positive population was on the opposite side (Supplemental Figure 1(f)). Notably, this organization was already established at 72 h, when aggregates are still spherical (Figure 6(a)). This observation suggested that XEN cells guide symmetry breaking by a local effect on the adjacent mESCs.

We speculated that this effect might be mediated by a basement membrane produced by the XEN cells. As established above, cells were polarized early during XEG development, prior to forming a columnar epithelium (Supplemental Figure 1(c) and (d)). This epithelium was supported by a basement membrane containing laminin and collagen (Figure 6(b) and (c)), which were mostly produced by the XEN cells (Supplemental Figure 8(a)). CellPhoneDB analysis of the scRNA-seq data supported the existence of laminin signaling between XEN-derived cells and multiple mESC-derived cell types (Supplemental Figure 8(b)). It has been demonstrated previously, for small aggregates of mESCs, that the presence of an extracellular matrix can be sufficient for polarization and lumen formation.^25,26,40^ Exposing gastruloids to a soluble basement membrane extract (Geltrex) did result in some, fragmented epithelia, if exposure was started after WNT activation (Supplemental Figure 8(c)). If exposure was started earlier, localized T-positive or SOX2-positive compartments were not formed, possibly due to unknown factors in the basement membrane extract that interfered with WNT activation. The results of the Geltrex experiments are consistent with the notion that the basement membrane provided by the XEN cells plays a role in epithelium formation.

To test whether XEN cells produced other, diffusible factors that were also involved, we grew gastruloids in medium conditioned by XEN cells (Figure 6(d)). We observed that the gastruloids did not elongate and had a T-positive cell population that was restricted to the center of the aggregate. We hypothesized that the WNT inhibitor DKK1, which is expressed in XEN cells (Figure 6(e); Supplemental Figure 9(a)), might be one of those factors. In vivo, DKK1 is expressed by the AVE and limits the growth of the primitive streak,^51^ together with the NODAL antagonists CER1 and LEFTY1, which are not expressed in XEGs (Figure 6(e), Supplemental Figure 4(c)). Growing gastruloids in the presence of DKK1 resulted in a round morphology, with the T-positive cells confined to the center, as observed for XEN-conditioned medium (Figure 6(f), Supplemental Figure 9(b)). Factors limiting the primitive streak expansion in vivo are also known to preserve the anterior part of the epiblast and are thereby necessary for proper ectoderm domain differentiation.^52^ Thus, we wanted to explore, if DKK1 could have a similar role in XEGs and bias differentiation toward the ectodermal lineage. Growing XEGs with the DKK1 inhibitor WAY-262611^53^ led to XEGs with elongated shapes but without epithelial structures (Figure 6(g), Supplemental Figure 9(c)). Since growing XEGs without CHIR resulted in similar epithelial structures as in regular XEGs (Supplemental Figure 9(d)), XEN cells likely suppressed pre-existing, low-level endogenous WNT activity.^5,54^

Finally, we noticed that XEN-derived cells highly expressed BMP2 (Supplemental Figure 9(a)) and that several of the dorsal markers expressed in XEGs are induced by BMP signaling (Supplemental Table 3). Cell-cell communication analysis of our scRNA-seq data supported the presence of BMP2 signaling between XEN-derived cells and multiple mESC-derived cell types, including neural ectoderm-like cells (Supplemental Figure 9(e)). Thus, XEN-derived cells might also contribute to the dorsal characteristics of the neural progenitor cells in XEGs.

All combined, our experiments suggest that XEN cells guide symmetry-breaking by local inhibition of cell differentiation into a T-positive population. Diffusible factors, including DKK1, and the presence of a basement membrane both seem to contribute to the formation of the neuroepithelial structures.

## Discussion

In this study we explored how the interaction between embryonic and extraembryonic cells in a multicellular in vitro system can lead to the formation of neuroepithelial tissue. While the neuroepithelial cells resembled in vivo neural progenitors transcriptionally, their organization was lacking, compared to embryonic neural tissue. In vivo, the neural tube forms via two distinct mechanisms.^55^ During primary neurulation, the main part of the neural tube is formed by the folding of the neural plate, an epithelial sheet of neural ectoderm cells. Secondary neurulation, which gives rise to the most posterior part of the neural tube, works differently: mesenchymal cells condense to an epithelial rod which cavitates to form a tube.^56,57^ The two parts of the tube are then connected during junctional neurulation.^58^ While we did not observe cell rearrangements characteristic of primary neurulation, the rosette formation seen in XEGs was reminiscent of secondary neurulation,^55^ which gives rise to the posterior neural tube. We could successfully differentiate XEGs further toward neural organoids that showed layered organization reminiscent of the developing spinal cord, which derives from the posterior neural tube.

The recently reported Trunk-Like Structures (TLS),^38^ another gastruloid-derived model system, produce neural tube-like tissues, together with mesodermal tissue resembling somites. Notably, TLS are formed exclusively from mESCs and are grown in 5% Matrigel from 96 h onward. Interestingly, the majority of neural tube cells in TLS had dorsal characteristics, as we also observed in XEGs. It will be interesting to explore, if the same mechanisms cause this phenomenon in both model systems. Another recent approach uses BMP4-treated ESCs as signaling centers to elicit neural tube-like tissues and other embryonic structures in untreated ESCs.^13^

The fact that the majority of XEN cells becomes VE-like in XEGs clearly shows that there are reciprocal interactions between the co-differentiating, embryonic and extraembryonic cells. This observation supports the notion that such interactions are necessary for proper development, as previously observed in vivo.^12,59^ Recently, tissue-level organization has been achieved in vitro by exogenous induction of relevant signaling pathways.^16,39,60^ XEN cells represent a potential alternative way to augment existing developmental in vitro systems, by providing a basement membrane and extraembryonic signaling inputs.

Finally, with their large diversity of cell types, XEGs could be a starting point for developing more complex models containing all three germ layers as well as extraembryonic cells. Specifically, the CD31 positive endothelial cells observed in the neural organoids obtained from XEGs might be able to form a vascular network, if additional signaling cues are given.^61^

In conclusion, in this study we showed how the gastruloid system can be used to explore complex heterotypic cell-cell interactions.

## Methods

### Experimental methods

#### Cell culture

All cell lines were routinely cultured in KO DMEM medium (Gibco) supplemented with 10% ES certified FBS (Gibco), 0.1 mM 2-Mercaptoethanol (Sigma-Aldrich), 1 × 100 U/mL penicillin/streptomycin, 1× MEM Non-Essential Amino Acids (Gibco), 2 mM L-glutamine (Gibco), 1000 U/mL mouse LIF (ESGRO). Cells were passaged every other day and replated in tissue-culture treated dishes coated with 0.2% gelatin. E14 mouse ES cells were provided by Alexander van Oudenaarden. The *Sox1*^GFPiresPac^ mouse ES cell line was created by Mario Stavridis and Meng Li in the group of Austin Smith^62^ and provided by Sally Lowell. XEN and XEN-*eGFP* were provided by Christian Schröter.^42^ All cell lines were regularly tested for mycoplasma infection. The ES-mCherry-GPI cell line was obtained by introducing an mCherry-GPI transgene in the *Pdgfra*^H2B-GFP^ cell line, provided by the group of Anna-Katerina Hadjantonakis.^63^

### Differentiation

#### Gastruloids

The gastruloid differentiation protocol was adapted from Van den Brink et al.^4^ ES cells were collected from tissue-culture treated dishes by trypsinization, gentle trituration with a pipet and centrifugation (1200 rpm, 3 min). After collection, cells were resuspended in 2 mL of freshly prepared, prewarmed N2B27 medium: DMEM/F12 (Life technologies) supplemented with 0.5 × N2 supplement (Gibco), 0.5 × B27 supplement (Gibco), 0.5 mM L-glutamine (Gibco), 1 × 100 U/mL penicillin/streptomycin (Gibco), 0.5 × MEM Non-Essential Amino Acids (Gibco), 0.1 mM 2-Mercaptoethanol (Sigma-Aldrich). Cells were counted to determine the cell concentration. For gastruloids, 200 ES cells were seeded in 40 µL of N2B27 in each well of a round-bottom low-adherence 96-well plate. 48 h after seeding, 150 µL of prewarmed N2B27 supplemented with 3 µM of GSK3 inhibitor (CHIR99021, Axon Medchem) was added to each well. 72 h after seeding, 150 µL of medium was removed from each well and replaced by 150 µL of preheated N2B27. Gastruloids were collected at 96 h after seeding and fixed with 4% paraformaldehyde (PFA, Alfa Aesar) overnight at 4°C.

For the experiments with gastruloids grown in Geltrex, cell aggregates were cultured with medium supplemented with 5% LDEV-Free, hESC-Qualified, reduced growth factor Geltrex (Gibco) from 24, 48, or 72 h until the end of the protocol. At 96 h, gastruloids were washed twice with PBS supplemented with 1% BSA, then fixed with 4% PFA overnight at 4°C.

#### XEN Enhanced Gastruloids (XEGs)

ES and XEN cells were collected from tissue-culture treated dishes by trypsinization, gentle trituration with a pipet and centrifugation (1200 rpm, 3 min). After collection, cells were resuspended in 2 mL of fresh and prewarmed N2B27 medium. Cells were counted to determine cell concentration. For XEGs, several ratios of XEN and ES cells were tested (1:1, 1:2, 1:3, 1:4, 1:5) and compared with the regular gastruloid condition (0:1). The total number of cells was fixed at 200. Over two separate experiments, the proportion of organoids showing T staining and epithelial structures was quantified (total number of embryonic organoids 1:1 = 179, 1:2 = 143, 1:3 = 143, 1:4 = 140, 0:1 = 130) and the optimal ratio was determined to be 1:3 (Supplemental Figure 1(e) and (f)). A total of 200 cells (150 ES cells and 50 XEN cells) was seeded in 40 µL of N2B27 in each well of a round-bottom low-adherence 96-well plate. 48 h after seeding, 150 µL of prewarmed N2B27 supplemented with 3 µM of GSK3 inhibitor (CHIR99021, Axon Medchem) was added to each well. 72 h after seeding, 150 µL of medium was removed from each well and replaced by 150 µL of prewarmed N2B27. XEGs were collected at 96 h after seeding and fixed with 4% PFA overnight at 4°C.

For the experiment of XEGs grown without GSK3 inhibitor, cells were seeded as usual. At 48 h, 150 µL of preheated N2B27 was added to each well. At 72 h, 150 µL of medium was removed from each well and replaced by 150 µL of prewarmed N2B27. XEGs were collected at 96 h after seeding.

For the smFISH control experiments, XEN cells were seeded at low density in N2B27 medium. At 48 h the medium was replaced by prewarmed N2B27 supplemented with 3 µM of GSK3 inhibitor. 72 h after seeding, the medium was replaced with prewarmed N2B27. Cells were fixed at 96 h with 4% PFA for 1 h at 4°C.

#### Neural differentiation

For neural differentiation, a protocol for creating cerebral organoids was adapted from Lancaster et al.^35^ Instead of collecting XEGs at 96 h, the medium was replaced by cerebral organoid differentiation medium: DMEM-F12 (Life technologies), Neurobasal (Gibco), 0.5 × B27 supplement containing vitamin A (Gibco), 0.5 × N2 supplement (Gibco), 2.5 µM/mL Insulin, 2 mM L-glutamine (Gibco), 0.5 × MEM-Non-Essential Amino Acids (Gibco), 1 × 100 U/mL penicillin-streptomycin and 0.05 mM 2-Mercaptoethanol (Sigma-Aldrich). At 168 h, aggregates were collected and transferred, with fresh medium, into 10 cm dishes on an orbital shaker installed in the incubator (85 rpm). Aggregates were grown until 192 h (8 days) during which medium was refreshed every other day until collection. Collected aggregates were fixed with 4% PFA for 48 h at 4°C.

### Signaling experiments

In the signaling experiments with XEGs, aggregates were treated between 72 and 96 h with either LDN193189 (BMPi, 100 nM, Reagents Direct), a potent BMP pathway inhibitor, Purmorphamine (1 µM, STEMCELL Technologies), a small molecule agonist of the hedgehog pathway, Retinoic acid (RA, 100 nM, Sigma-Aldrich) or DMSO (0.1% final concentration, Sigma Aldrich) as a vehicle control. For this experiment, the XEGs were allowed to grow for an additional 48 h before fixation (144 h total growth) and preparation for staining (see Immunostaining).

DKK1 signaling pathways perturbation was performed in two ways, using DKK1 (Sigma-Aldrich) for activation in gastruloids and Way262611 (Sigma-Aldrich) for inhibition in XEGs. Gastruloids and XEGs were seeded according to the usual protocols. At 24 h, 40 µL of N2B27 supplemented with various concentration of DKK1 or Way262611 respectively, were added to each well. Next steps of the protocol were performed using N2B27 supplemented with DKK1 or Way262611. Aggregates were fixed at 96 h with 4% PFA overnight at 4°C.

### Immunostaining

#### Fixation and blocking

After collection, gastruloids and XEGs were fixed in 4% PFA at 4°C overnight. Tissue resulting from the cerebral organoid protocol was fixed under the same conditions, but for 48 h. After fixation, samples were washed three times in washing solution (PBS, 1% bovine serum albumin (BSA)) and incubated at 4°C in blocking buffer (PBS, 1% BSA, 0.3% Triton-X-100) for a minimum of 16 h. Samples for smFISH were washed three times in PBS after fixation and stored in 70% ethanol at 4°C. To stain E14 cells for pluripotency markers, cells in suspension were fixed for 30 min in 4% PFA at 4°C, washed three times in washing solution at RT and incubated in blocking buffer for 1 h at 4°C.

#### Whole-mount immunolabeling and clearing

Immunolabeling and clearing of gastruloids and XEGs were based on the protocol described by Dekkers et al.^64^ Briefly, after fixation and blocking, samples were incubated with primary antibodies at 4°C overnight on a rolling mixer (30 rpm) in organoid washing buffer (OWB) (PBS, 2% BSA, 0.1% Triton-X-100) supplemented with 0.02% sodium dodecyl sulfate (SDS), referred to as OWB-SDS. The following primary antibodies were used: rat anti-SOX2 (1:200, 14-9811-82, Thermo Fisher Scientific), goat anti-T (1:200, sc-17745, Santa Cruz Biotechnology), goat anti-T (1:100, AF2085, R&D systems), mouse anti-DAB2 (1:100, 610464, BD Biosciences). The next day, samples were washed three times for 2 h in OWB-SDS at RT, followed by incubation with secondary antibodies (donkey anti-goat Alexa Fluor 488 (1:200, A-11055, Thermo Fisher Scientific), donkey anti-rat Alexa Fluor 488 (1:200, A-21208, Thermo Fisher Scientific), donkey anti-goat Alexa Fluor 555 (1:200, A-21432, Thermo Fisher), donkey anti-mouse Alexa Fluor 555 (1:200, A-31570, Thermo Fisher Scientific), chicken anti-rat Alexa Fluor 647 (1:200, A-21472, Thermo Fisher Scientific)) and 4′,6-diamidino-2-phenylindole (DAPI, 1 µg/mL, Merck) in OWB-SDS at 4°C overnight on a rolling mixer (30 rpm), protected from light. Finally, samples were washed three times for 2 h in OWB-SDS at RT. Clearing was performed by incubation in fructose-glycerol clearing solution (60% vol/vol glycerol, 2.5 M fructose) for 20 min at RT. Samples were imaged directly after clearing or stored at 4°C in the dark.

#### Cryo-sectioning and immunolabeling of sections

Prior to cryosectioning, fixed and blocked samples were incubated sequentially in sucrose solutions (10%, 20%, and 30%) for 30 min (gastruloids and XEGs) or 2 h (neural organoids) at 27°C, and embedded in optimal cutting temperature (OCT) compound. Samples in OCT were placed on dry ice for rapid freezing, and stored at −80°C prior to cryosectioning. Samples were cut to cryosections (10 µm thickness) using a cryostat (Thermo Fisher Scientific, USA) and cryosections were placed on poly-L-lysine coated glass slides (Merck). The slides were stored directly at −80°C. For immunofluorescence staining, slides were thawed and rinsed with PBS for 10 min at RT to dissolve the OCT. Subsequently, slides were incubated overnight at 4°C with the following primary antibodies diluted in blocking buffer: rat anti-SOX2 (1:200, 14-9811-82, Thermo Fisher Scientific), goat anti-T (1:200, sc-17745, Santa Cruz Biotechnology), mouse anti-N-cadherin (1:200, 33-3900, Thermo Fisher Scientific), rabbit anti-E-cadherin (1:200, 3195, Cell Signaling Technology), rabbit anti-PAX6 (1:100 (cerebral organoids) or 1:200 (gastruloids, XEGs), 42-6600, Thermo Fisher Scientific), mouse anti-NKX6.1 (1:200, F55A12, Developmental Studies Hybridoma Bank), rabbit anti-NKX6.1 (1:200, HPA036774, Merck), mouse anti-TUJ1 (1:200, 801202, BioLegend), goat anti-PAX2 (1:200, AF3364, R&D Systems), goat anti-TBX6 (1:200, AF4744, R&D Systems), mouse anti-ASCL1 (1:200, 14-5794-80,, Thermo Fisher Scientific), rat anti-CTIP2 (1:200, ab18465, abcam), rabbit anti-CD31 (1:50, ab28364, Abcam), rabbit anti-GATA6 (1:200, PA1-104, Thermo Fisher Scientific), goat anti-GATA6 (1:200, AF1700, R&D Systems), rabbit anti-Laminin (1:200, PA1-16730, Thermo Fisher Scientific), mouse anti-OCT4 (1:200, MA1-104, Thermo Fisher Scientific), rabbit anti-ZO-1 (1:200, 40-2200, Thermo Fisher Scientific), mouse anti-aPKC (1:200, sc-17781, Santa Cruz Biotechnology), anti-Msx1 (1:200, PA5-35227, Thermo Fisher Scientific), mouse anti-Nrcam (1:200, S364-51, Thermo Fisher Scientific) and goat anti-Collagen IV (1:200, NBP1-26549, Novus Biological). The next day, the slides were washed twice for 10 min in PBS at RT. Subsequently, the slides were incubated with secondary antibodies (donkey anti-goat Alexa Fluor 488 (1:200, A-11055, Thermo Fisher Scientific), donkey anti-rat Alexa Fluor 488 (1:200, A-21208, Thermo Fisher Scientific), donkey anti-goat Alexa Fluor 555 (1:200, A-21432, Thermo Fisher), donkey anti-mouse Alexa Fluor 555 (1:200, A-31570, Thermo Fisher Scientific), chicken anti-rat Alexa Fluor 647 (1:200, A-21472, Thermo Fisher Scientific), donkey anti-rabbit Alexa Fluor 647 (1:200, A-31573, Thermo Fisher Scientific)) and DAPI (1 µg/mL, Merck) in blocking buffer for 4 h at 4°C, and washed three times for 10 min at RT. Slides were mounted in ProLong™ Gold Antifade Mountant (Thermo Fisher Scientific) and imaged after 24–48 h.

#### Immunolabeling of E14 cells

After fixation and blocking, E14 cells were incubated with the following primary antibodies in blocking buffer overnight at 4°C: rat anti-SOX2 (1:200, 14-9811-82, Thermo Fisher Scientific) and mouse anti-OCT4 (1:200, MA1-104, Thermo Fisher Scientific). The next day, cells were washed three times in washing solution for 5 min at RT and incubated with secondary antibodies (donkey anti-rat Alexa Fluor 488 (1:200, A-21208, Thermo Fisher Scientific) and donkey anti-mouse Alexa Fluor 555 (1:200, A-31570, Thermo Fisher Scientific)) and DAPI (1 µg/mL, Merck) in blocking buffer for 3 h at 4°C. Finally, the cells were washed three times in washing solution for 5 min at RT and imaged directly.

### Single-molecule fluorescence in-situ hybridization (smFISH)

smFISH was performed as described previously.^65^ Briefly, samples were fixed with PFA and stored in 70% ethanol, as described above. Custom designed smFISH probes for *Dab2, Fst, Hhex, Spink1, Wnt4, Wnt8a, Fgf8, Cer1, Dkk1 and Bmp2* (BioCat, Supplemental Table 5), labeled with Quasar 570, CAL Fluor Red 610, or Quasar 670, were incubated with the samples overnight at 30°C in hybridization buffer (100 mg/mL dextran sulfate, 25% formamide, 2X SSC, 1 mg/mL E.coli tRNA, 1 mM vanadyl ribonucleoside complex, 0.25 mg/mL BSA; Thermo Fisher Scientific). Samples were washed twice for 30 min at 30°C with wash buffer (25% formamide, 2X SSC). The wash buffer was supplemented with DAPI (1 μg/mL) in the second wash step. All solutions were prepared with RNAse-free water. Finally, the samples were mounted in ProlongGold (Life Technologies) and imaged when hardened (sections) or immediately (ibidi dishes). All components are from Sigma-Aldrich unless indicated.

### Imaging

Fixed and stained samples were imaged on a Nikon Ti-Eclipse epifluorescence microscope equipped with an Andor iXON Ultra 888 EMCCD camera and dedicated, custom-made fluorescence filter sets (Nikon). Primarily, a 10×/0.3 Plan Fluor DLL objective, a 20×/0.5 Plan Fluor DLL objective, or a 40×/1.3 Super Fluor oil-immersion objective (Nikon) were used. To image complete sections of neural organoids, multiple adjacent fields of view were acquired and combined using the tiling feature of the NIS Elements software (Nikon). Z-stacks were collected of whole-mount gastruloids and XEGs with distances of 10 μm between planes. For smFISH measurements, z-stacks were collected with a distance of 0.2 μm between planes in four fluorescence channels (DAPI, Quasar 570, CAL Fluor Red 610, Quasar 670) using a 100×/1.45 Plan Apo Lambda oil (Nikon) objective. Time lapses to observe the formation of epithelial structures were performed 24 and 48 h after cell seeding, on XEGs grown from the mCherry-GPI ES cell line. XEGs were transferred to a glass-bottom μ-Slide imaging chamber (ibidi) and imaged every 30 min for 24 h with a Nikon Eclipse Ti C2+ confocal laser microscope (Nikon, Amsterdam, The Netherlands), equipped with lasers at wavelengths 408, 488, and 561, an automated stage and perfect focus system at 37°C and 5% CO_2_. Images were acquired with a Nikon 20× Dry Plan Apo VC NA 0.75 objective. To track SOX1 expression in gastruloids and XEGs during the 24 h growth after the GSK3 inhibitor pulse, 72 h gastruloids and XEGs grown from the *Sox1*^GFPiresPac^ ES cell line were transferred to a glass-bottom μ-Slide imaging chamber (ibidi) and imaged every 40 min for 24 h, while temperature and CO_2_ levels were maintained at 37°C and 5%, respectively, by a stage top incubator (INUG2-TIZW-SET, Tokai Hit) mounted on the Nikon Ti-Eclipse epifluorescence microscope.

### Single-cell RNA-seq library preparation and sequencing

For each replicate, 96 pooled gastruloids and 96 pooled XEGs were collected from a round-bottomed low-adherence 96-well plate in 15 mL Falcon tubes and pelleted by gentle centrifugation (500 rpm for 2 min). No final aggregate was excluded from the collection. After washing with cold PBS, samples were resuspended in N2B27. Cells were then dissociated by 5 min incubation in TrypLE (Gibco) and gentle trituration with a pipet, centrifuged and resuspended in 1 mL of cold N2B27. Cells were counted to determine cell number and viability. For the first replicate, ES-mCherry-GPI were spiked in at a frequency of 5%. For the second replicate, E14 cells were collected from culture dishes and incubated for 30 min at 4°C with CITE-seq cell hashing^66^ antibody Ab_CD15 (1:200) (Biolegend). XEN*-eGFP* were collected from culture plates and incubated for 30 min at 4°C with CITE-seq cell hashing antibody Ab_CD140 (1:200) (Biolegend). In the gastruloid sample, labeled E14 cells were spiked in at a frequency of 5%, whereas in the XEG sample labeled E14 and XEN-*eGFP* were spiked in, both at a frequency of 5%. High viability of the cells in all samples was confirmed before 10X library preparation. Single-cell RNA-seq libraries were prepared using the Chromium Single Cell 3′ Reagent Kit, Version 3 Chemistry (10× Genomics) according to the manufacturer’s protocol. CITE-seq libraries were prepared according to the CITE-seq protocol from New York Genome Center version 2019-02-13. Libraries were sequenced paired end on an Illumina Novaseq6000 at 150 base pairs.

## Computational methods

### Analysis of single-cell RNA-sequencing data

#### Single-cell RNA-seq data pruning and normalization

Cells with a low number of transcripts were excluded from further analysis based on the histograms in Supplemental Figure 3(a) (count <1300 for replicate 1 of the XEG experiment and count <2300 for the other datasets). Genes expressed in less than two cells (across merged replicates) were excluded from further analysis. The final XEG dataset contains 14,286 genes and 4591 or 6857 cells for replicate 1 or 2, respectively. The gastruloid dataset contains 14,384 genes and 4233 or 8363 cells per replicate. The two datasets were normalized using the scran R-package (V 1.10.2^67^). Gene variabilities were calculated (improvedCV2, scran) for each replicate separately, after excluding ribosomal genes [Ribosomal Protein Gene Database, http://ribosome.med.miyazaki-u.ac.jp/], exogenously expressed genes and the cell hashing antibodies. The 10% most highly variable genes (HVG) were selected based on variability *p*-values.

#### Dimensionality reduction

For each of the two datasets, the two replicates were batch corrected with the fast mutual nearest neighbors (MNN) method implemented in the scran R-package,^68^ using the union of the 10% HVG of the two replicates and log-transformed normalized counts with *d* = 120 (number of principal components) and *k* = 50 (number of nearest neighbors). For dimensionality reduction, a uniform manifold approximation and projection (UMAP) was calculated on the batch corrected data using the R-package UMAP (V 0.2.3.1) with *n* = 50, min_dist = 0.7 and using the cosine distance measure.

#### Identification of spike-in cells

Cells with any expression of mCherry were annotated as ES (mCherry+). The remaining spike-in cells, E14 (CD15+) and XEN spike-in (CD140+) (see Single-cell RNA-seq library preparation and sequencing), could not be determined by the expression level of the antibody alone. We therefore chose to assign spike-ins based on clusters. For each of the two datasets, a shared nearest neighbor graph was constructed from the batch corrected data (see Dimensionality reduction) with scran using *k* = 20 and *d* = 30. Louvain clustering was performed on the constructed graphs with the R-package igraph (V1.2.4.1), which resulted in eight clusters for XEGs and seven clusters for gastruloids (see Supplemental Figure 3(c)). We identified three out of the eight clusters in XEGs based on literature markers and spike-in gene expression. One cluster out of these three was mainly comprised of mESCs, due to high Ab_CD15 expression and mCherry positive cells. Cells that had an expression of Ab_CD15 >50 and were part of this cluster were considered spiked-in E14 and annotated as E14 (CD15+). The other two clusters were both eGFP positive, where one of them had a higher Ab_CD140 expression and was thus annotated as XEN spike-in (Ab_CD140+). The second cluster was annotated as XEN derived (Ab_CD140−). Similarly, for gastruloids, one of the seven clusters was comprised of mainly mESCs based on literature markers and spike-in gene expression. Cells that had an expression of Ab_CD15 >100 and were part of this cluster were considered spiked-in E14 and annotated as E14 (CD15+).

#### Analysis of cell cycle and stress-related genes

For each of the two datasets, cell cycle analysis was performed with the scran package using the cyclone function^69^ on the normalized counts. Cells in G2M phase were distributed evenly across all clusters and thus the clustering was not biased by cell cycle. No other separate cluster that consisted entirely of cell cycle related cells appeared.

For the analysis of stress-related genes, a list of known stress genes^70^ was used to calculate the average standardized expression per cell based on normalized counts. Stress-related genes were mainly found within the spike-in cells and there was no other separate cluster that consisted entirely of highly stressed cells.

#### Mapping to in vivo datasets

Our datasets were mapped to three different in vivo datasets.

##### Pijuan-Sala et al

dataset.

The Pijuan-Sala et al. dataset,^37^ which was downloaded from https://content.cruk.cam.ac.uk/jmlab/atlas_data.tar.gz, consists of nine timepoints from E6.5 to E8.5. The data was normalized by size factors provided by the authors. Cells with no cell type assignment were excluded from further analysis. The 10% HVG were calculated (improvedCV2, scran package) on the remaining cells excluding sex genes, similar to Pijuan-Sala et al.’s method. Cells in the “mixed_gastrulation” cluster were also excluded. MNN mapping was applied to log-transformed normalized counts of the 10% HVG. First, in vivo timepoints were mapped to each other in decreasing order. Then, each of our four datasets was mapped separately to the combined Pijuan-Sala et al. dataset (MNN method with *d* = 120, *k* = 50). K-nearest-neighbor (knn) assignment was performed in the batch corrected principal component space. For each cell in our datasets, the 50 nearest neighbors in the in vivo dataset, based on Euclidean distances, were calculated. Each cell was assigned the most abundant cell type within the knn, if certain distance and confidence score conditions were met. This confidence score was calculated for each cell as the number of the most abundant cell type divided by the total number of neighbors (*k* = 50). A cell was annotated as “Not assigned” if either, the average distance to its nearest neighbor exceeded a certain threshold (determined by the long tail of the histogram of average distances for each of our datasets separately) or the assignment had a confidence score less than 0.5. Additionally, we placed cells in “Not assigned” if they were assigned to clusters with less than 10 cells, or to the cluster “Blood progenitors 2” (because this cluster did not show distinct expression of known literature markers). This resulted in 22 assigned clusters for XEGs and 15 assigned clusters for gastruloids. For each cell in our dataset we calculated the average and the standard deviation of the developmental age of the knn.

##### Nowotschin et al. dataset

The Nowotschin et al. dataset,^43^ which was downloaded from https://endoderm-explorer.com/, consists of six timepoints from E3.5 to E8.75. The data was normalized (scran) and the 10% HVG were calculated (improvedCV2, scran package). First, MNN was applied to the Nowotschin et al. dataset in increasing order of the timepoints (using log-transformed normalized counts of the 10% HVG, *d* = 150, *k* = 50). Then, XEN cells from our XEG dataset (XEN spike-ins (CD140+) and XEN derived (CD140−)) were mapped to the MNN-corrected Nowotschin et al. dataset. Knn assignment was performed as described above and resulted in seven assigned clusters.

##### Delile et al. dataset

The Delile et al. dataset,^36^ which was downloaded from https://github.com/juliendelile/MouseSpinalCordAtlas, consists of five timepoints from E9.5 to E13.5. Cells that had a cell type assignment of “Null” or “Outlier” were excluded from further analysis. The data was normalized (scran) and the 10% HVG were calculated. First, MNN was applied to the Delile et al. dataset in order of increasing timepoints (log-transformed normalized counts of the 10% HVG, *d* = 120, *k* = 50). Then, we mapped neural ectoderm-like clusters, identified through the mapping to the Pijuan-Sala et al. dataset (“Rostral neurectoderm,” “Caudal neurectoderm,” “Spinal cord,” and “Forebrain/Midbrain/Hindbrain”) to the MNN corrected Delile et al. dataset separately for each of our replicates. Knn assignment was performed as described above and resulted in three clusters for XEGs and three clusters for gastruloids.

#### Differential expression analysis

For the differential expression test between “spike-in XENs” and “XENs in XEGs” a Welch *t*-test (implemented in findMarkers, scran R package) was conducted on the normalized log-transformed counts. The test was performed on XEGs from replicate 2. “spike-in XENs” were chosen as the 100 cells with highest Ab_CD140 expression and “XENs in XEGs” were the 100 cells with lowest Ab_CD140 expression within the XEN identified cells.

For the differential expression test between XEGs and gastruloids, a negative binomial regression was performed (R package edgeR V 3.24.3^71^). Based on the knn assignment to the Pijuan-Sala et al. dataset, all “neural ectoderm-like” clusters (“Rostral neurectoderm,” “Caudal neurectoderm,” “Spinal cord,” and “Forebrain/Midbrain/Hindbrain”) were extracted from our four datasets (XEGs: 975 cells in replicate 1 and 357 cells in replicate 2; gastruloids: 2134 cells in replicate 1 and 2106 cells in replicate 2). Raw counts were used for the regression with these four subsets as dummy variables and a variable corresponding to the total number of counts per cell. *p*-Values were obtained for the contrast between XEGs and gastruloids using the average regression coefficients among variables of both replicates.

Similarly, for the differential expression test of the “Spinal cord” in XEGs, a negative binomial regression was used. Cells were excluded from the test if either their cell type occurred in less than 10 cells per replicate, or if the cells were annotated as “Not assigned,” leaving a total of 13 cell types (7742 cells) to be considered. For each cell type and each replicate a dummy variable was created and a variable corresponding to the total number of counts per cell. Then, *p*-values were obtained for the contrast between the average regression coefficients of the two replicates of the “Spinal cord” cluster and the average regression coefficients of all other variables considered in the test.

For all differential expression tests *p*-values were adjusted for multiple hypothesis testing with the Benjamini-Hochberg method.

#### Sub-clustering of neural ectoderm-like cells

Neural ectoderm-like cells (“Rostral neurectoderm,” “Caudal neurectoderm,” “Spinal cord,” and “Forebrain/Midbrain/Hindbrain”) were extracted from the XEG data sets for Figure 4(e) to (g) and Supplemental Figure 5(d). A curated list of genes that are dorsoventral axis markers in the developing neural tube^28,36,38^ was used for all analysis steps (see Figure 4(f) for the complete list). First, replicates were integrated with MNN using *d* = 5 and *k* = 20. The UMAP was created from the MNN corrected subspace with 20 nearest neighbors, min_dist = 0.3 and cosine metric. K-means clustering was performed on the MNN corrected subspace using Euclidean distances and five centers.

For Supplemental Figure 5(d), neural ectoderm-like cells were extracted from XEG and gastruloid data sets. As before, only genes listed in the heatmap in Figure 4(f) were used for all analysis steps. MNN mapping was performed using *d* = 15 and *k* = 20 in the following sequence: XEG replicate 2, XEG replicate 1, gastruloid replicate 2, and gastruloid replicate 1. UMAP and clustering was performed as described before. To correct for the difference in the number of cells coming from the four samples, first, relative frequencies for the five sub-clusters were calculated per sample. These frequencies were then normalized by dividing by the sum of relative frequencies for a specific sub-cluster.

#### Cell-cell interaction analysis with CellPhoneDB

CellPhoneDB^49^ was applied to the raw counts of replicate 2 of the XEG data set. All mouse gene names were converted to human gene names with the *biomaRt* R package. All clusters, assigned through the mapping to the Pijuan-Sala et al. dataset, were used. Finally, results containing the ligands of interest (BMP2, BMP4, and LAM) were extracted. For each pair of cell types with significant communication (*p*-value <0.05), the expression of all significant ligand-receptor pairs was summed. The expression of a ligand-receptor pair was taken to be the average of ligand and receptor expression.

### Image analysis

Image stacks of whole-mount immunostained gastruloids and XEGs, and images of immunostained sections were pre-processed by background subtraction (rolling ball, radius: 50 pixels = 65 μm (10× objective), 32 μm (20× objective), or 16 μm (40× objective)) in the channels that showed autofluorescent background using ImageJ.^72^ When background subtraction in images of sections did not result in proper removal of autofluorescent background signal, the Enhance Local Contrast (CLAHE) tool was used in ImageJ.^72^ smFISH image stacks were pre-processed by applying a Laplacian of Gaussian filter (σ = 1) to the smFISH channels using scikit-image (v0.16.1).^73^ For all image stacks, a maximum projection was used to obtain a 2D representation. To show a single object per image, images were cropped around the object of interest.

## Supplementary Material

Supplementary material

Supplementary material

Supplementary material

Supplementary material

Supplementary material

Supplementary material

Supplementary material

Supplementary material

Supplementary material

Supplementary material
