# Supplementary material for: A gastruloid model of the interaction between embryonic and extra-embryonic cell types
Source: J Tissue Eng. 2022 Jun 11;13:20417314221103042. doi: 10.1177/20417314221103042 (PMC9189523; doi:10.1177/20417314221103042)
Supplement: Supplementary material [file sj-pdf-1-tej-10.1177_20417314221103042.pdf]

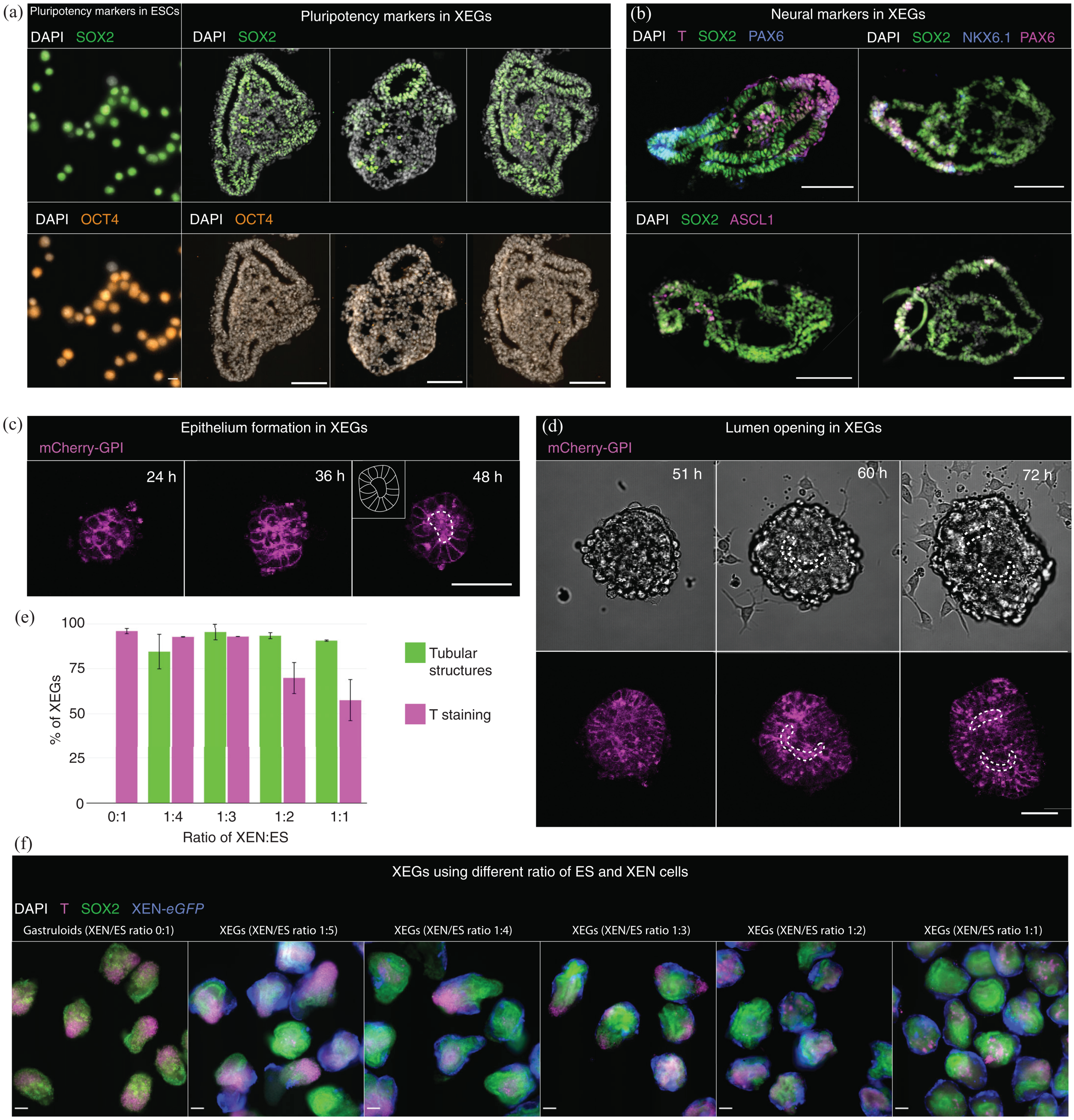

Supplementary Fig. 1 | Optimization and characterization of XEGs. a, Expression of SOX2 and OCT4 (immunostaining) in cultured ESCs (left, scale bar: 10 µm) and sections of XEGs at 96 h (right, scale bars: 100 µm). b, Expression of T, SOX2, PAX6, ASCL1 and NKX6.1 (immunostaining) in sections 96 h XEGs. Scale bars: 100 µm. c-d, Live-cell imaging of morphological changes in XEGs grown from mCherry-GPI expressing mESCs. mCherry-GPI is localized to the cell membrane. In all images, a single z-plane is shown. Scale bars: 50 µm. c, Rosette formation. The center of the rosette is indicated by a dashed line. Inset: tracing of cell outlines. See also Supplementary Video 1. d, Cavitation of rosettes. The top row shows the brightfield channel, the bottom row shows the mCherry channel. Dashed lines indicate the opening lumen. See also Supplementary Video 2. e, Average fraction of aggregates showing epithelial structures and T staining at 96 h for different starting ratios of ESCs and XEN cells (n = 2 experiments, error bars show standard deviation). f, SOX2 and T expression in gastruloids and XEGs with different starting ratios of ESCs and XEN cells (z-projection of whole mount immunostaining). Scale bars: 100 µm. a, b, f, Cell nuclei were stained with DAPI.

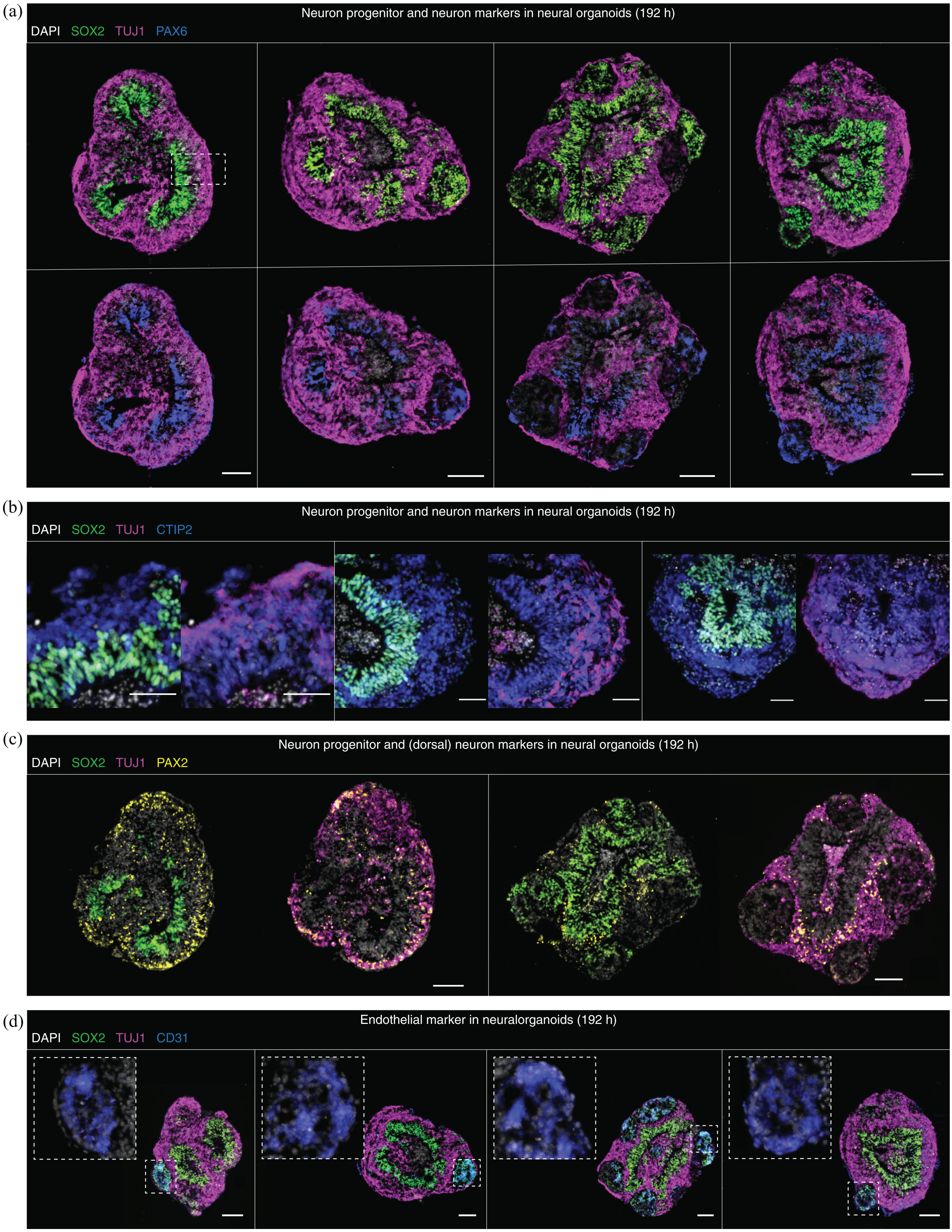

Supplementary Fig. 2 | Developing spinal cord markers are expressed in XEGs further differentiated with a cerebral organoid protocol. a-d, Immunostaining of sections of XEGs on day 8 after cell seeding. a, TUJ1, a neuron marker, and SOX2 (top) and PAX6 (bottom), neural progenitor markers. The dashed box highlights an example of layered organization reminiscent of the ventricular and mantle zones in the developing spinal cord. b, TUJ1, SOX2 and CTIP2, a neuron marker. c, TUJ1, SOX2 and PAX2, a marker of dorsal neurons in the developing spinal cord. d, TUJ1, SOX2 and CD31, an endothelial marker. Insets show clusters of cells positive for CD31. a-d, Cell nuclei were stained with DAPI. Scale bars: 100 µm.

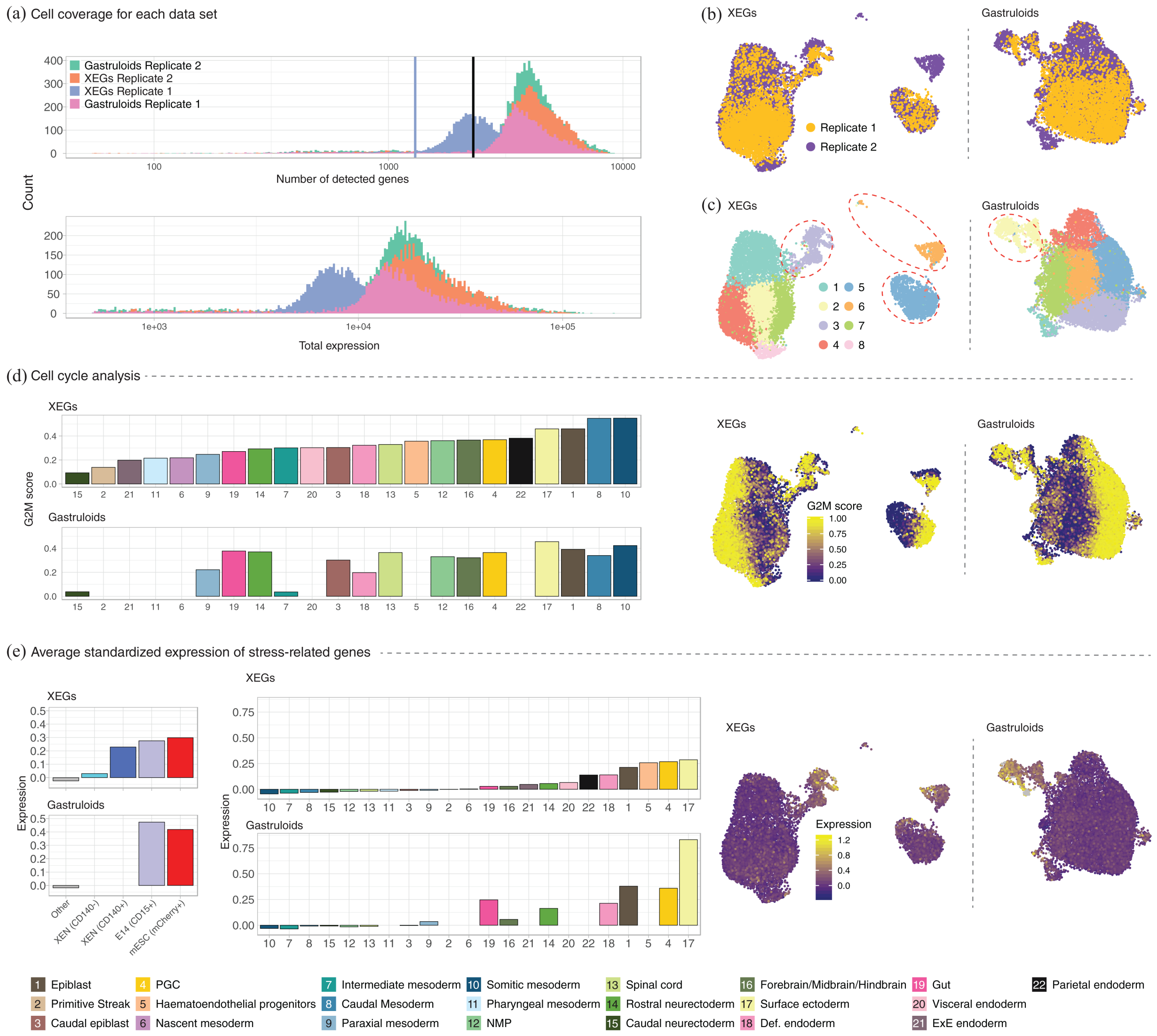

Supplementary Fig. 3 | Quality control of single-cell RNA-seq data. a, Top, number of detected genes per cell in each replicate; the blue line indicates a quality control threshold for XEGs from replicate 1 and the black line for the remaining datasets. Bottom, total expression per cell for each dataset. b, UMAP of cells in XEGs and gastruloids, colored by replicate. c, UMAP of cells in XEGs and gastruloids, colored by Louvain clustering. The encircled clusters contain the spiked-in cells. d, Left, average G2M scores for each cell type. Right, UMAPs of cells in XEGs and gastruloids colored by G2M score. e, Left, average standardized expression of stress-related genes in spike-in cells. Middle, expression of stress-related genes by cell type. Right, UMAPs of cells in XEGs and gastruloids with expression of stress-related genes indicated by color. b-e, UMAPs contain both replicates, batch corrected.

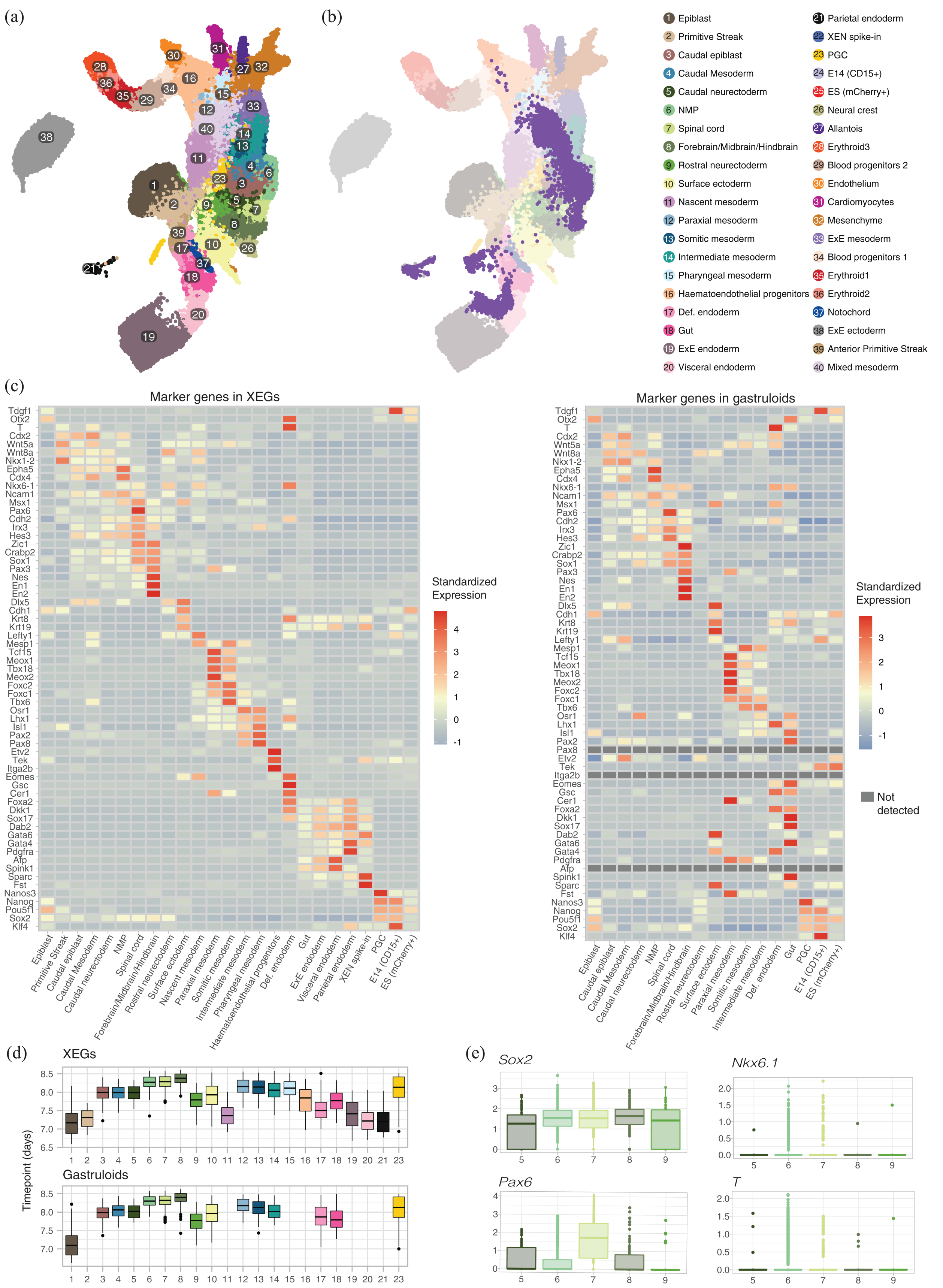

Supplementary Fig. 4 | Single-cell RNA-seq resolves cell type diversity in XEGs and gastruloids. a, UMAP of the Pijuan-Sala et al.37 dataset with cell types indicated by color. b, MNN mapping of XEG cells from replicate 2 (opaque color) to the Pijuan-Sala et al. dataset (pale colors), as an example for the mapping procedure. c, Heat map of standardized expression of genes associated with mouse embryonic development in XEGs and gastruloids. References describing the in vivo expression of the genes are given in Supplementary Table 1. d, Developmental age of cell types based on mapping to in vivo data. e, Expression of Sox2, Pax6 and Nkx6.1 and T in XEGs, as measured by single-cell RNA-seq.

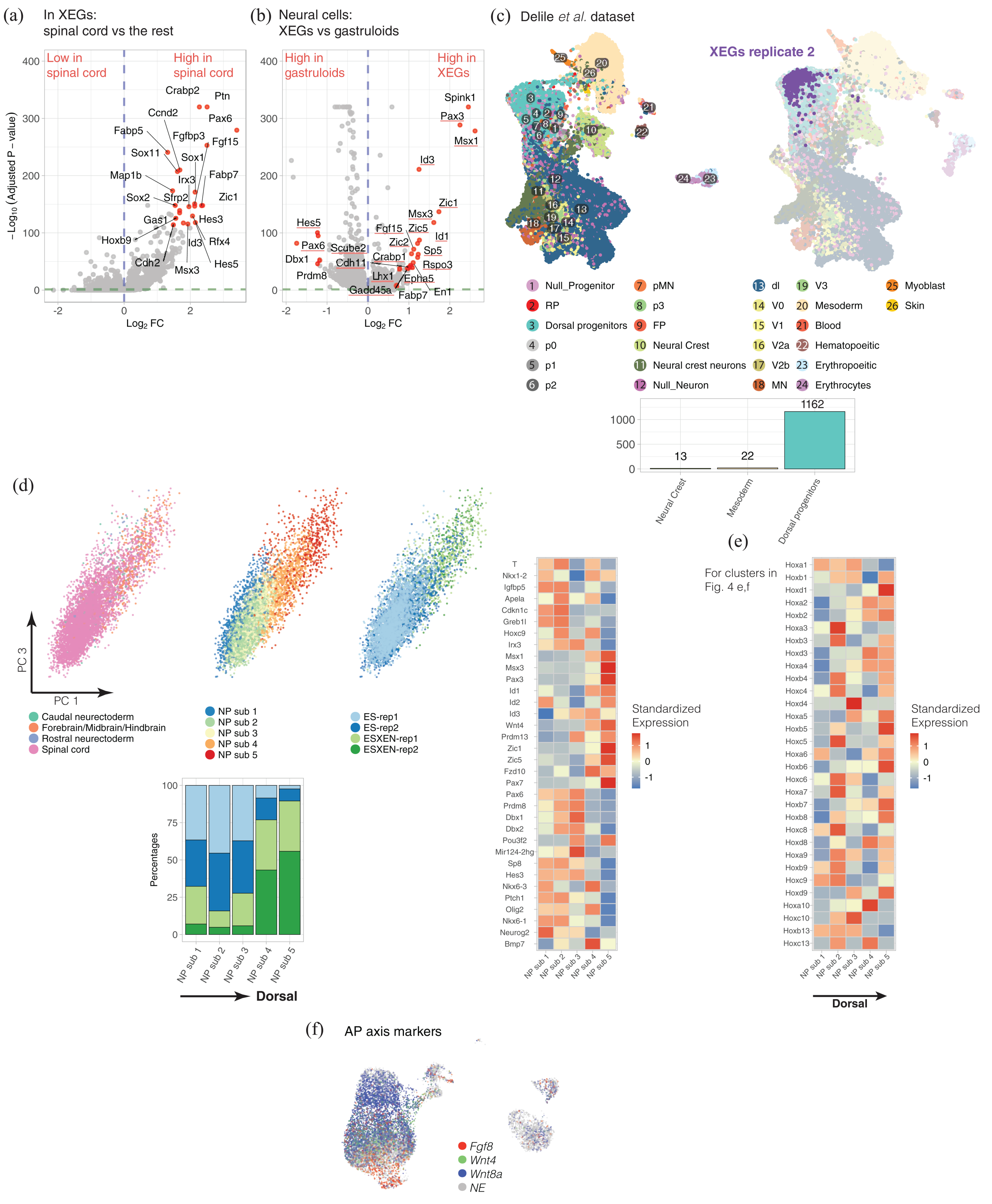

Supplementary Fig. 5 | Neuroepithelial cells in XEGs are biased towards a dorsal expression profile. a, Gene expression differences between cells classified as "spinal cord" and all other cells in XEGs (fold-change vs p-value). Named genes are expressed in the neural tube according to previous studies (Supplementary Table 2). b, Gene expression differences between neural ectoderm-like cells in gastruloids and XEGs (fold-change vs p-value). Underlined genes are expressed in the dorsal part of the neural tube according to previous studies (Supplementary Table 3). c, Left, UMAP of the cells in the Delile et al. dataset36, colored by cell type. Right, MNN mapping of cells classified as "spinal cord" in replicate 2 XEGs (opaque color) to the Delile et al. dataset (pale colors), as an example of the mapping procedure. Bottom: Absolute cell type frequency as a result of MNN mapping. d, Top row: Principal component analysis of neural ectoderm-like cells from all 4 data sets (XEGs and gastruloids) after integration. Colors from left to right: Cell types based on mapping to in vivo data, sub-clusters and data sets. Bottom: Relative frequency (percentage) of data sets in each sub-cluster. Right: Heatmap of standardized expression of dorsoventral markers in the sub-clusters shown in the PCA plot. e, Heatmap of standardized expression of Hox genes in neural ectoderm-like sub-clusters shown in Fig. 4e. f, UMAP of cells in XEGs with log-expression of the genes Wnt4, Wnt8a and Fgf8 indicated by colors. The UMAP shows both replicates, batch corrected.

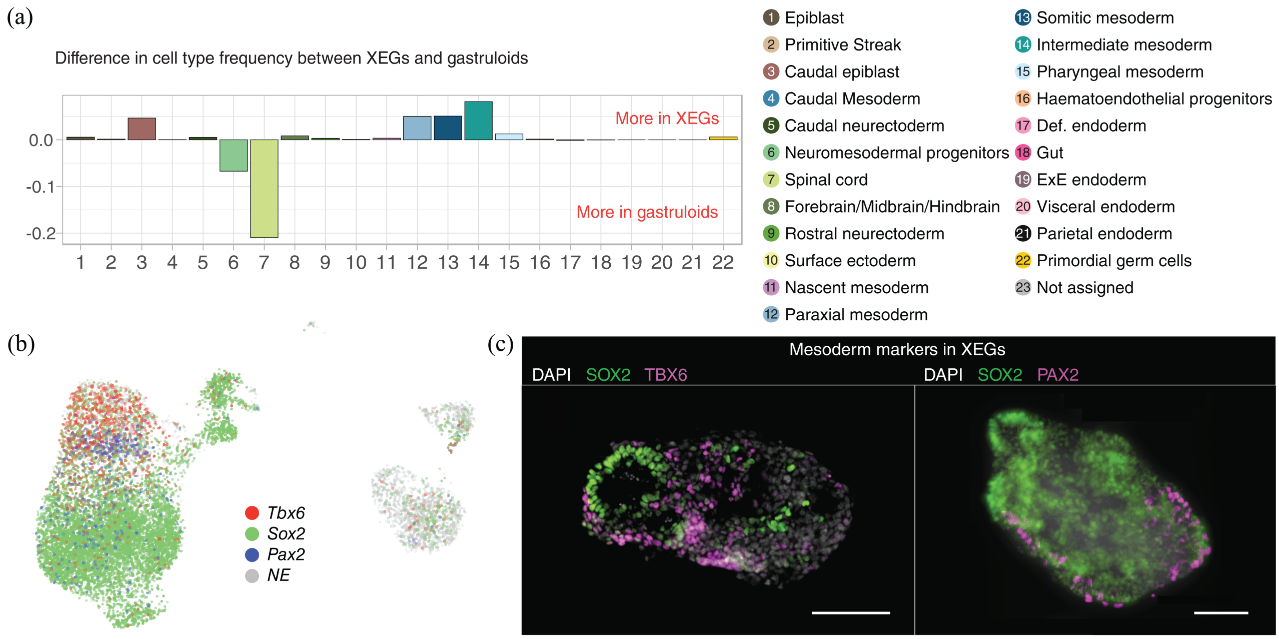

Supplementary Fig. 6 | XEGs and gastruloids differ in cell type distribution. a, Differences between relative frequencies of cell types in XEGs and gastruloids. b, UMAP of cells in XEGs (both replicates, batch corrected) with log expression of Tbx6, Sox2 and Pax2 indicated by color. c, Expression of mesoderm markers. Left, TBX6 expression in a 96 h XEG (immunostaining of a section). Right, PAX2 expression in a 96 h XEG (wholemount immunostaining). Scale bars: 100 µm. Nuclei were stained with DAPI.

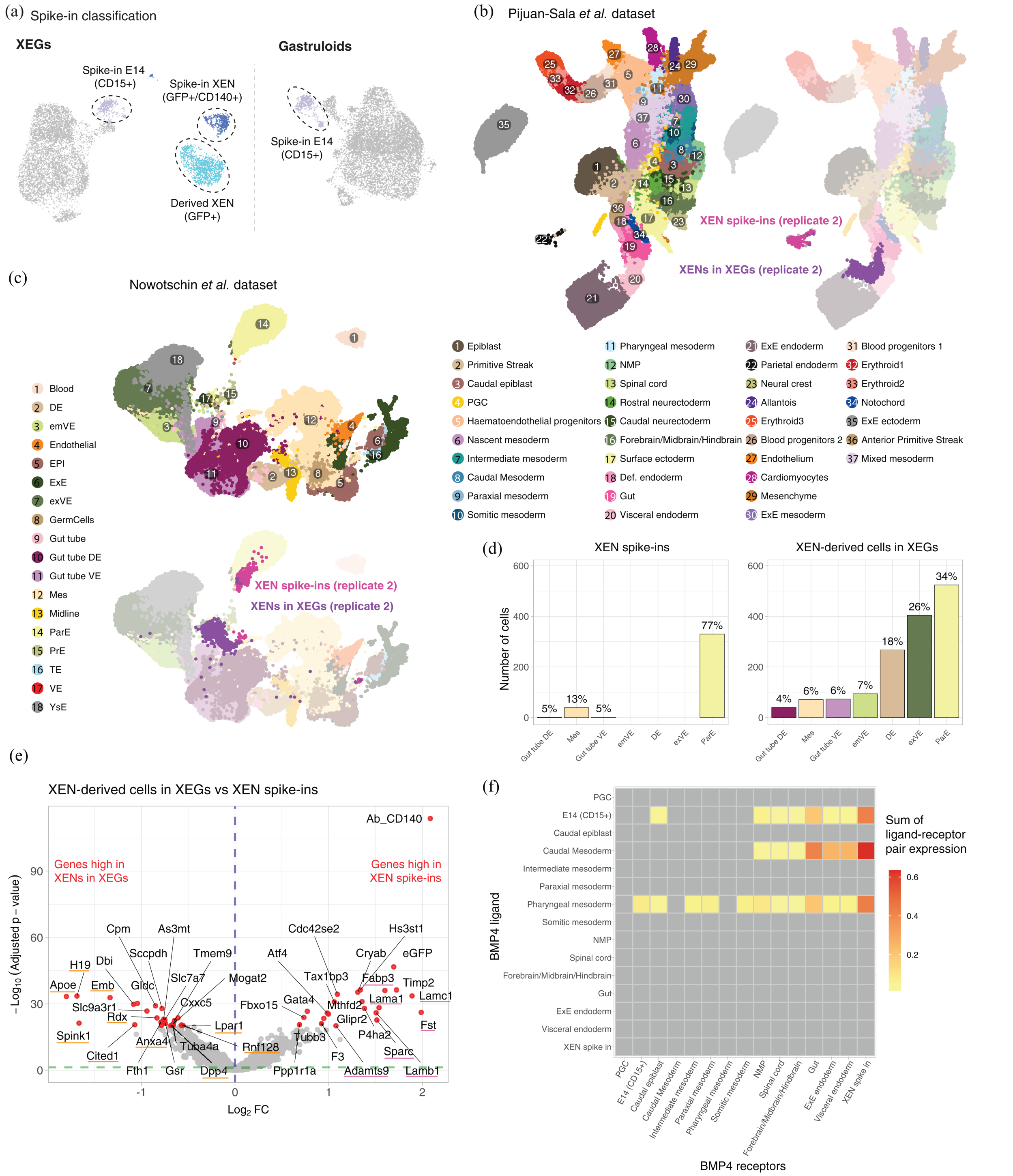

Supplementary Fig. 7 | Most XEN-derived cells become visceral endoderm-like in XEGs. a, UMAP of cells in XEGs and gastruloids with spiked-in cells and XEN derived cells highlighted by color and circled (replicate 2). b, c, UMAPs of the Pijuan-Sala37 or Nowotschin43 dataset, respectively. XEN spike-ins and XEN-derived cells from XEG replicate 2 (opaque colors) are mapped to the in vivo datasets (pale colors). d, Cell type frequencies of XEN spike-ins and XEN derived cells in XEGs, resulting from knn assignments based on the mapping in (c). e, Gene expression differences between XEN spike-ins and XEN-derived cells in XEGs (fold-change vs p-value). Orange and pink lines indicate genes with PE-like and VE-like identity, respectively (see Supplementary Table 4). f, Sum of expression of BMP4 ligand-receptor pairs for cell types with significant communication identified by CellPhoneDB analysis. DE: definitive endoderm, emVE: embryonic visceral endoderm, EPI: epiblast, ExE: extraembryonic ectoderm, exVE: extraembryonic visceral endoderm, Mes: mesoderm, ParE: parietal endoderm, PrE: primitive endoderm, TE: trophectoderm, VE: visceral endoderm, YsE: yolk sac endoderm.

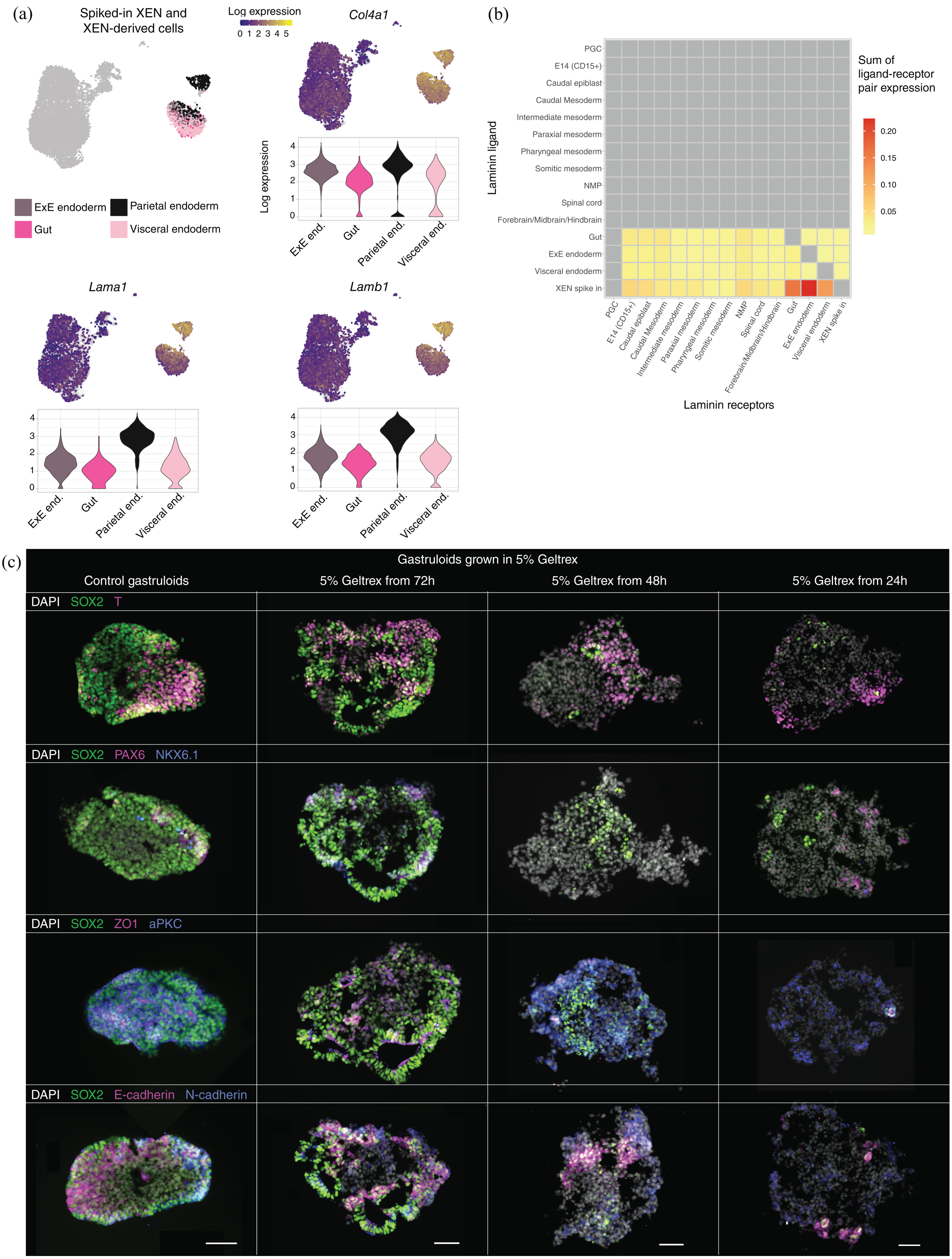

Supplementary Fig. 8 | Basement membrane components produced by XEN cells, play a role in epithelia formation. a, Expression of genes in spiked-in XEN cells and XEN-derived cells in XEGs. Top left, UMAP with spiked-in XEN cells and XEN-derived cells colored by cell type (gut, parietal endoderm (parietal end.), embryonic VE (visceral end.) or extraembryonic VE (ExE end.)). Expression of basement membrane components collagen IV (Col4a1, top right), laminin alpha 1 (Lama1, bottom left) and laminin beta 1 (Lamb1, bottom right). UMAPs indicate log expression by color and contain both replicates, batch corrected. A violin plot of log expression in XEN-derived cell types is shown below the UMAP for each gene. b, Sum of expression of Laminin ligand-receptor pairs for cell types with significant communication identified by CellPhoneDB analysis. c, Expression of SOX2, T, PAX6, NKX6.1, ZO-1, αPKCα, E-cadherin and N-cadherin in 96 h gastruloids grown in 5% Geltrex from 24 h, 48 h or 72 h onwards (immunostaining of cryosections). Scale bars: 50 μm. Cell nuclei were stained with DAPI.

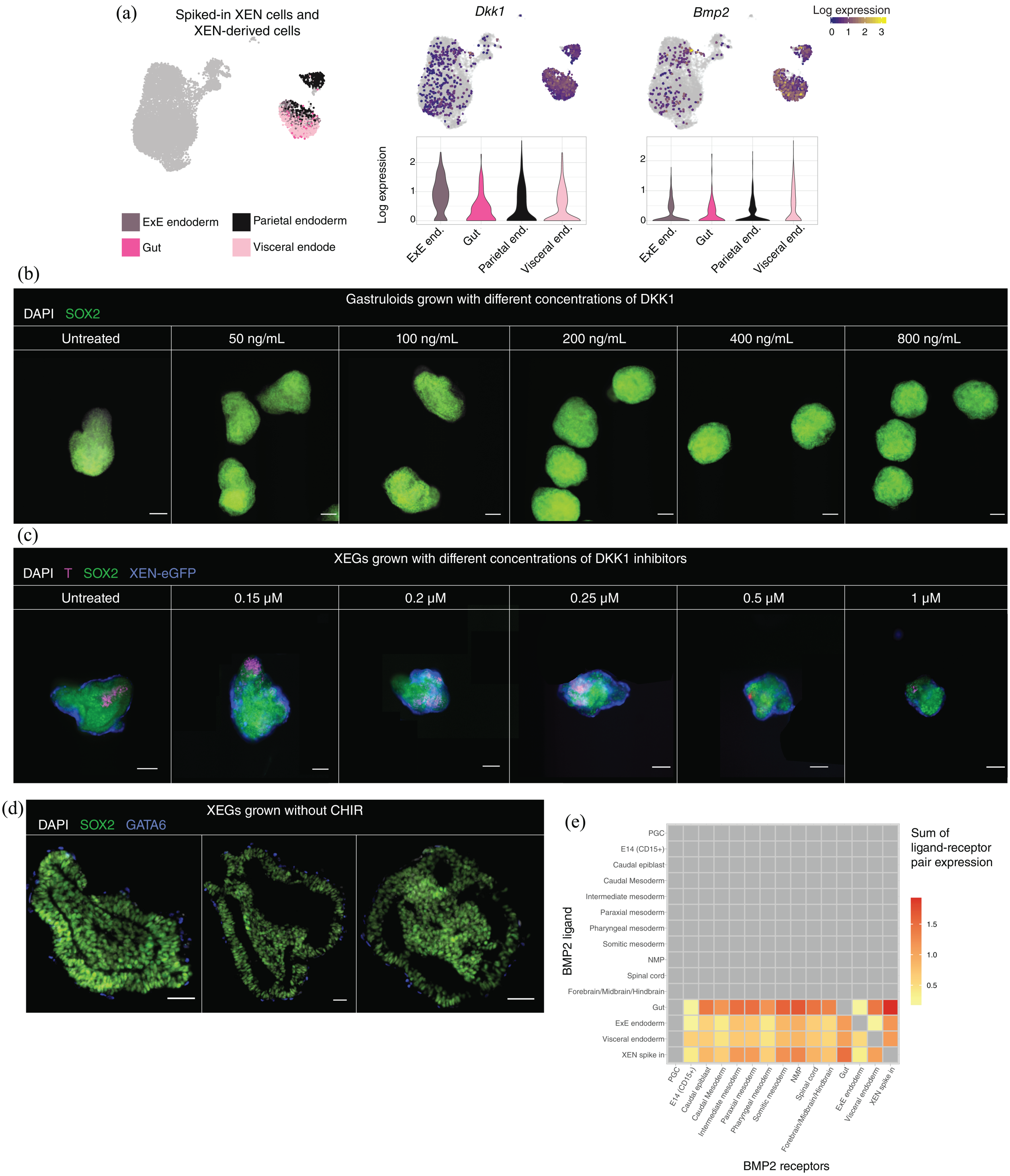

Supplementary Fig. 9 | The WNT inhibitor DKK1, expressed exclusively by XEN cells and XEN-derived cells, plays a role in epithelia formation. a, Expression of genes in spiked-in XEN cells and XEN-derived cells. Left, UMAP with spiked-in XEN cells and XEN-derived cells colored by cell type (gut, parietal endoderm (parietal end.), embryonic VE (visceral end.) or extraembryonic VE (ExE end.)). Right, expression of signaling factors Dkk1 and Bmp2. UMAPs indicate log-expression by color and contain both replicates, batch corrected. A violin plot of log expression in XEN-derived cell types is shown below the UMAP for each gene. b, Expression of SOX2 in 96 h gastruloids treated with various concentrations of DKK1 between 24 h and 96 h (wholemount immunostaining). Scale bars: 100 µm. c, Expression of SOX2 and T in 96 h XEGs treated with various concentrations of DKK1 inhibitor WAY-262611 between 24 h and 96 h (wholemount immunostaining). d, Expression of SOX2 in XEGs grown without CHIR (immunostaining of cryosections). No specific T staining could be detected (data not shown). XEN cells were localized by expression of GATA6. Scale bars: 50 µm. e, Sum of expression of BMP2 ligand-receptor pairs for cell types with significant communication identified by CellPhoneDB analysis.
