# Supplementary material for: A gastruloid model of the interaction between embryonic and extra-embryonic cell types
Source: J Tissue Eng. 2022 Jun 11;13:20417314221103042. doi: 10.1177/20417314221103042 (PMC9189523; doi:10.1177/20417314221103042)
Supplement: Supplementary material [file sj-docx-2-tej-10.1177_20417314221103042.docx]

**Supplementary Table 1 | Marker genes used for cell type annotation in the single-cell RNA-seq data**

**Supplementary Table 2 | Genes differentially expressed between spinal cord-like cells and the other cells types in XEGs.**

**
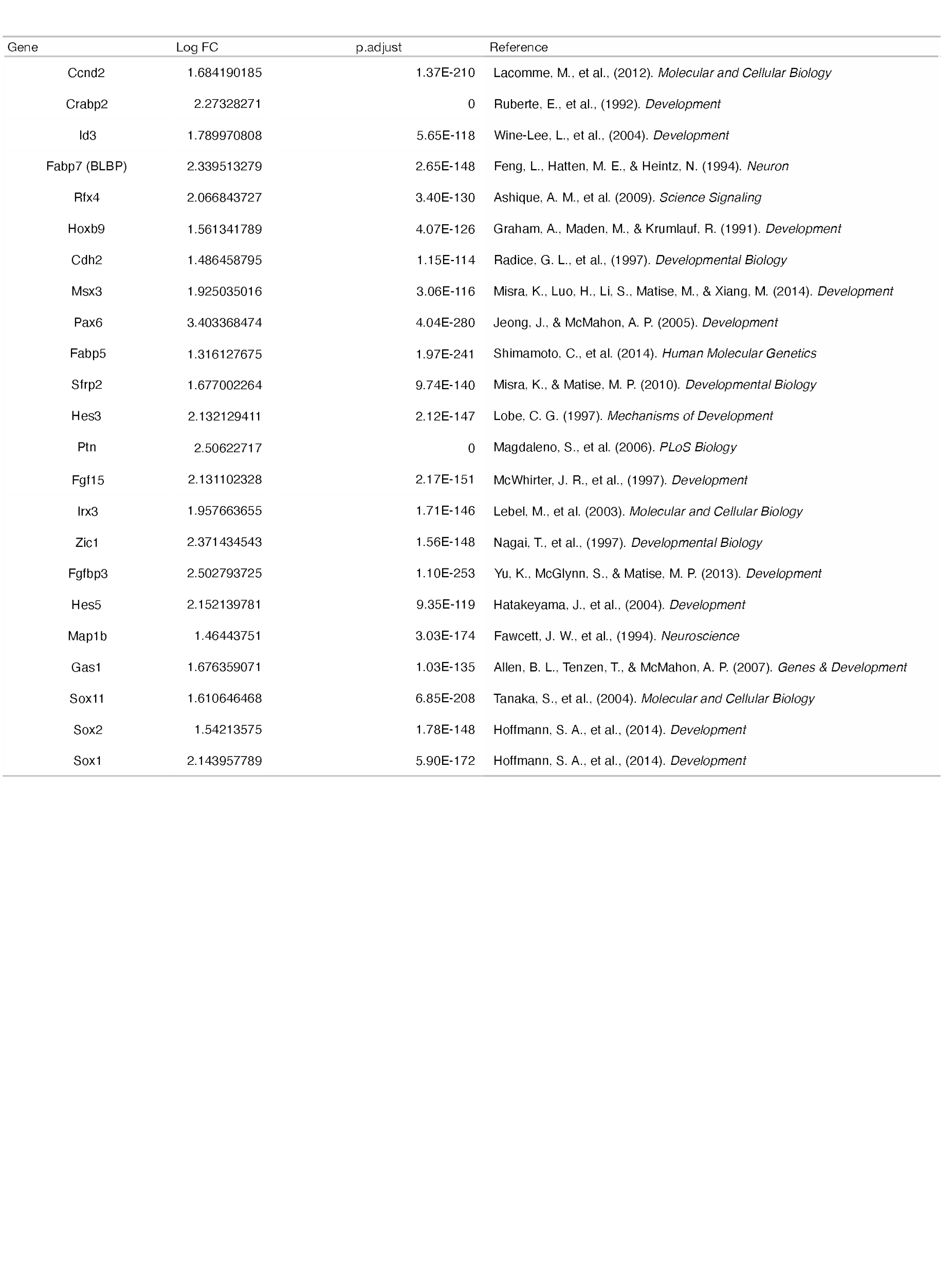
**

**Supplementary Table 3 | Genes differentially expressed between neural ectoderm-like cells in XEGs and gastruloids.**

**
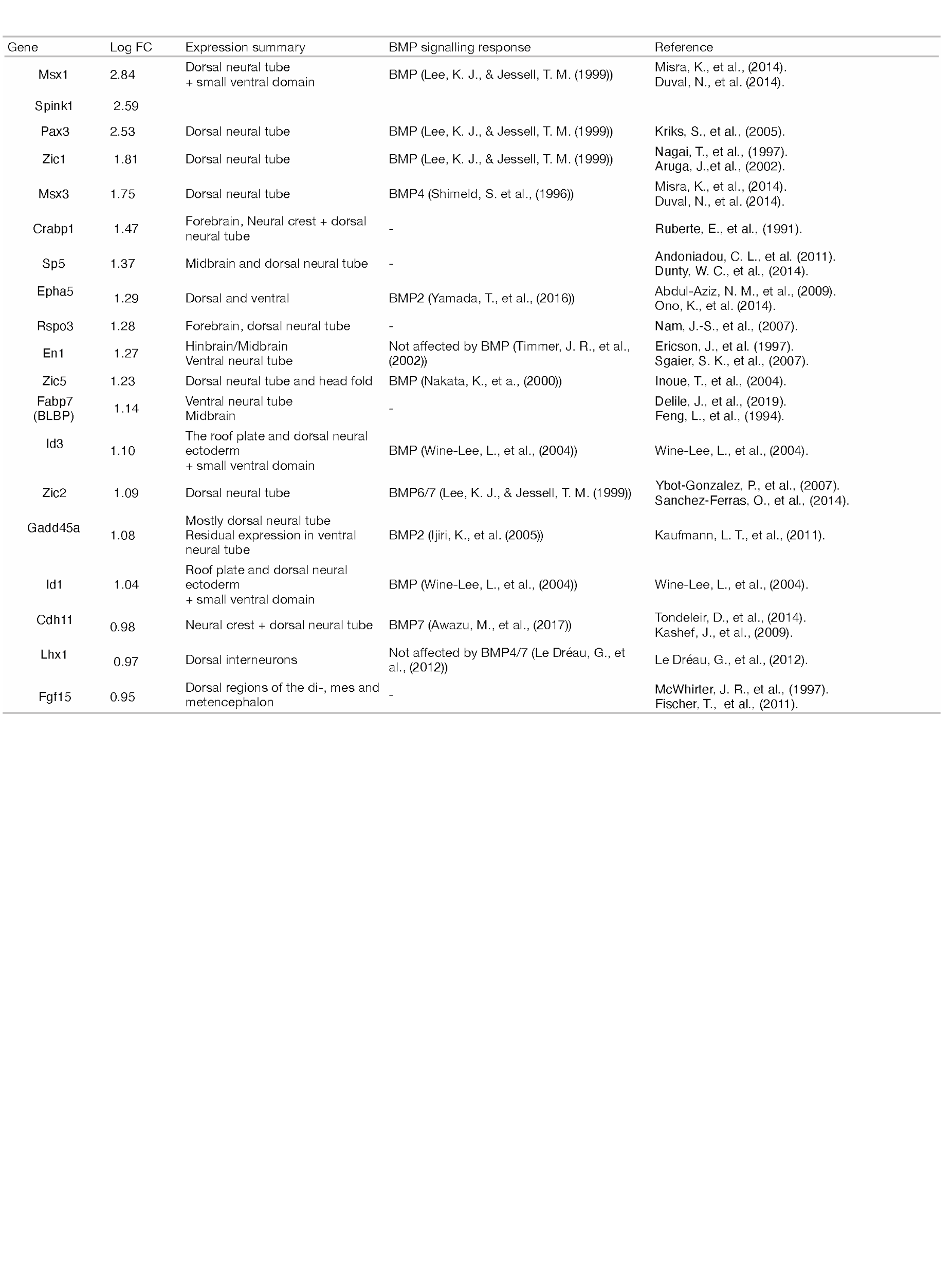
**

**Supplementary Table 4 | Genes differentially expressed between XEN-derived cells in XEGs and cultured XEN cells.**


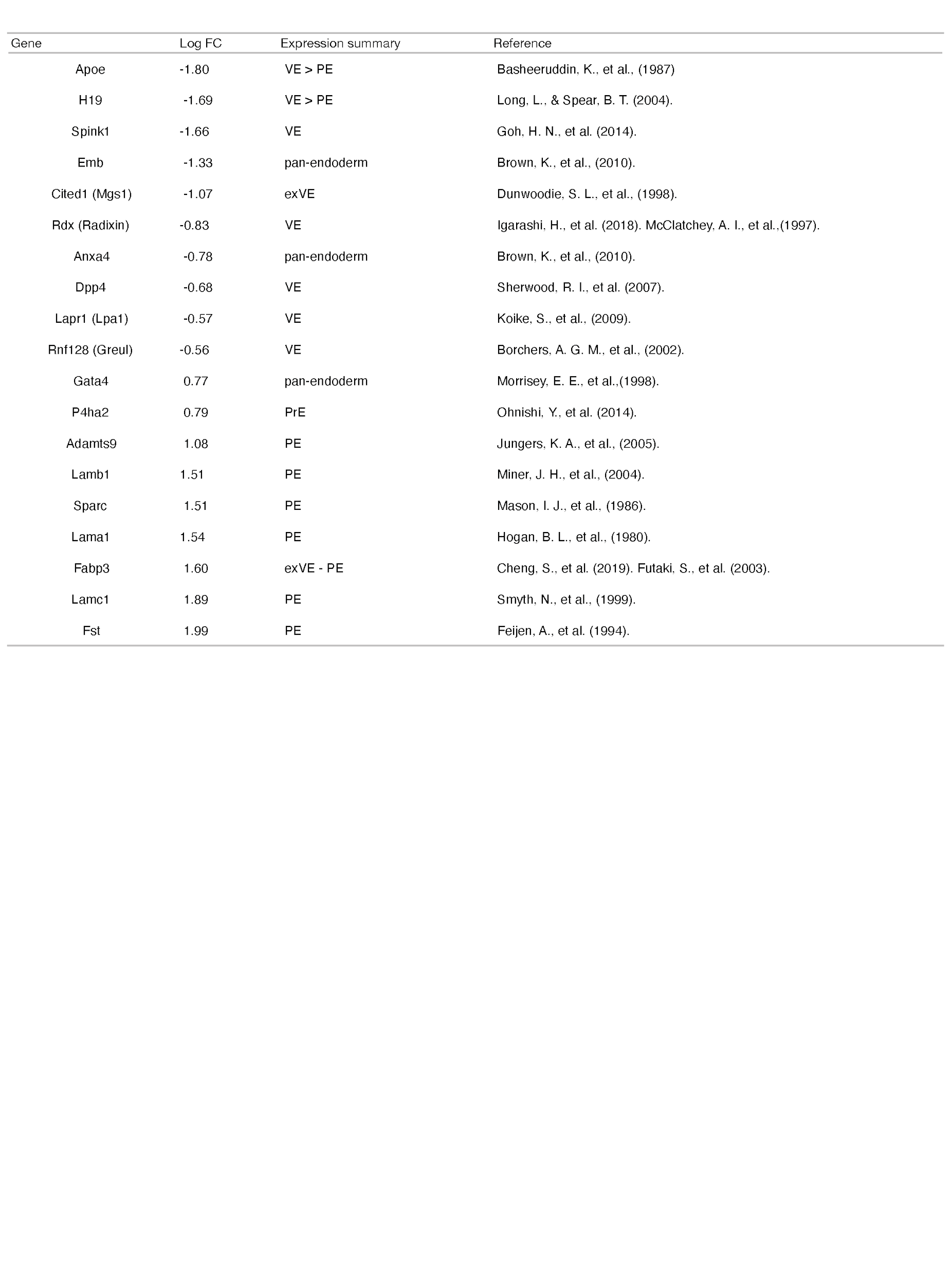


**Supplementary Table 5 | Single-molecule FISH probes**
